# Tubular-Cell-Derived Extracellular Vesicle miR-491-3p Aggravates Renal Ischemia–Reperfusion Injury by Inhibiting Macrophage SIRT1-Mediated Notch Intracellular Domain Deacetylation-Driven Ubiquitin–Proteasome Degradation

**DOI:** 10.34133/research.0929

**Published:** 2025-10-17

**Authors:** Peihan Wang, Bojun Li, Tianbao Song, Zefeng Wang, Zhen Yin, Baofeng Song, Sheng Zhao, Xiangjun Zhou, Weimin Yu, Fan Cheng, Wei Li

**Affiliations:** ^1^ Department of Urology, Renmin Hospital of Wuhan University, Wuhan, Hubei 430060, P.R. China.; ^2^ Department of Anesthesiology, Renmin Hospital of Wuhan University, Wuhan, Hubei 430060, P.R. China.

## Abstract

Renal ischemia–reperfusion injury triggers substantial inflammatory reactions, with renal tubular epithelial cells (TECs) and macrophages playing crucial roles. Extracellular vesicles (EVs) are key mediators of intercellular signaling. However, the precise roles and mechanisms by which TEC-derived EVs influence macrophage functions remain unclear. This study investigated how miR-491-3p within EVs from TECs modulates macrophage polarization, thus worsening renal inflammation and damage. In models that are in vitro and in vivo, EVs enriched with miR-491-3p from TECs subjected to hypoxia/reoxygenation were shown to promote M1 polarization in macrophages, enhancing inflammatory responses and inducing TEC apoptosis. Mechanistically, miR-491-3p directly targets sirtuin 1 (SIRT1) in macrophages, inhibiting SIRT1-mediated Notch intracellular domain (NICD) deacetylation, thereby regulating NICD stability. This regulation blocks F-box and WD repeat domain-containing 7 (FBXW7)–NICD binding, reduces NICD ubiquitination, and activates the Notch/nuclear factor kappa-light-chain-enhancer of activated B cells (NF-κB) pathway. Furthermore, Rab27a knockout, which limits EVs’ release, substantially reduces M1 macrophage polarization and renal tissue damage. These findings indicate that miR-491-3p from TEC-derived EVs targets SIRT1, inhibiting the deacetylation of NICD mediated by SIRT1, which subsequently prevents ubiquitin-mediated NICD degradation. This mechanism modulates the inflammatory phenotype of macrophages and promotes the inflammatory response, thereby worsening renal injury induced by ischemia–reperfusion injury and highlighting a potential therapeutic target.

## Introduction

Renal ischemia–reperfusion injury (IRI) is a important clinical issue commonly observed during kidney surgeries or procedures involving temporary renal artery occlusion [1,2]. Procedures such as partial nephrectomy, renal trauma repair, and artery clamping often cause ischemia and reperfusion injury [3]. IRI begins with ischemia-induced hypoxia disrupting homeostasis, worsened by oxidative stress, inflammation, endothelial dysfunction, and tubular epithelial cell (TEC) apoptosis during reperfusion [4]. These processes may lead to acute tubular necrosis, long-term renal dysfunction, or chronic kidney disease (CKD), with severe cases requiring dialysis [5,6]. The high incidence of IRI in these patients highlights the need for innovative strategies to reduce its effects, preserve renal function, and improve outcomes [7].

TECs, crucial for renal structure and function, are highly susceptible to IRI damage due to their high metabolic demands and stress vulnerability [8]. Inflammatory processes initiated by IRI severely compromise TECs, leading to dysfunction and cell loss [9]. Reperfusion causes damaged cells to release damage-associated molecular patterns and reactive oxygen species, activating inflammatory pathways like nuclear factor kappa-light-chain-enhancer of activated B cells (NF-κB) and toll-like receptors [1,10,11]. TEC damage is worsened by increased production of inflammatory mediators, such as tumor necrosis factor-α (TNF-α), interleukin-6 (IL-6), and interleukin-1β (IL-1β) [12]. Chronic inflammation disrupts TECs, promoting apoptosis via mitochondrial dysfunction and caspase activation [13]. TEC loss weakens tubular integrity, reduces renal function, and accelerates organ damage, highlighting inflammation’s role in TEC death during renal IRI. Notably, under such stress conditions, TECs can actively release extracellular vesicles (EVs) containing bioactive molecules, which may serve as key mediators of intercellular communication and modulate immune cell responses [14].

Macrophages, being the predominant immune response cells found in the kidney, are essential to the body’s innate immunity [15,16]. Macrophages identify tissue injury or infection, secreting cytokines like TNF-α, IL-6, and IL-1β and chemokines to amplify the inflammatory cascade [17]. Macrophages switch between M1 (damage) and M2 (repair) phenotypes, balancing damage and recovery [18]. Therefore, our study focused on macrophages. Increasing evidence indicates that TECs and macrophages communicate not only through soluble mediators such as cytokines and chemokines but also via EVs, particularly exosomes, which can transfer bioactive molecules between these cell types and modulate inflammatory responses [16].

Renal IRI involves TEC–macrophage interactions, with EVs mediating inflammation and tubular damage [19]. EVs carry RNA and other molecules, regulating inflammation and modulating macrophage function [20]. EV-mediated communication may coordinate inflammation and injury in renal ischemia–reperfusion (IR), highlighting its potential as a therapeutic target for TEC damage and renal recovery. This study investigated TEC-derived EVs’ role in macrophage inflammation and TEC damage during IRI.

## Results

### Macrophage-mediated inflammatory response aggravates renal injury in a mouse model of IR

A renal IRI model was established in mice, divided into 4 groups. Kidney samples were collected on days 1, 3, and 7, with a sham control group (Fig. S1A). Serum creatinine and urea nitrogen were measured to monitor renal function (Fig. S1B and C). Terminal deoxynucleotidyl transferase dUTP nick-end labeling (TUNEL) staining showed peak apoptosis on day 1, declining by days 3 and 7. Histological analysis revealed progressive renal injury. Macrophage infiltration and M1 polarization increased post-IRI, while M2 polarization was observed on days 3 and 7 (Fig. S1D and F to K). Although both M1 and M2 polarization occurred within a week, M1 polarization was predominant (Fig. S2D to G).

M1 markers CD86 and inducible nitric oxide synthase (iNOS) steadily increased post-IRI (Fig. S1E). Serum IL-1β, IL-6, and TNF-α maintained high levels throughout the week (Fig. S1L to N), reflecting sustained inflammation with M1 polarization [21]. Polymerase chain reaction (PCR) also showed elevated messenger RNA (mRNA) levels of inflammatory factors on day 1, indicating acute renal impairment (Fig. S2A to C). Despite repair, hematoxylin and eosin staining revealed ongoing damage from fibrosis and chronic inflammation, with apoptosis and extracellular matrix buildup worsening tissue disease [22]. In conclusion, renal injury, macrophage infiltration, and M1 polarization worsened during the first week post-IRI, with inflammatory cytokines maintained at high levels in serum and tissues.

Macrophage depletion via clodronate liposomes in a murine IRI model (administered 48 h before and on day 4 post-IRI) led to reduced F4/80-positive macrophages (Fig. S3A and C). Histological analysis showed less tubular damage and decreased kidney injury molecule-1 (Kim-1) expression (Fig. S3B, D, and E), while TUNEL staining revealed fewer apoptotic cells (Fig. S3B and F). Serum IL-1β, IL-6, and TNF-α were significantly reduced (Fig. S3G to I), suggesting that macrophages amplify inflammation and worsen renal damage post-IRI.

### EVs from renal tubules enhance M1 macrophage polarization, exacerbating IR kidney injury

**Fig. 1.**
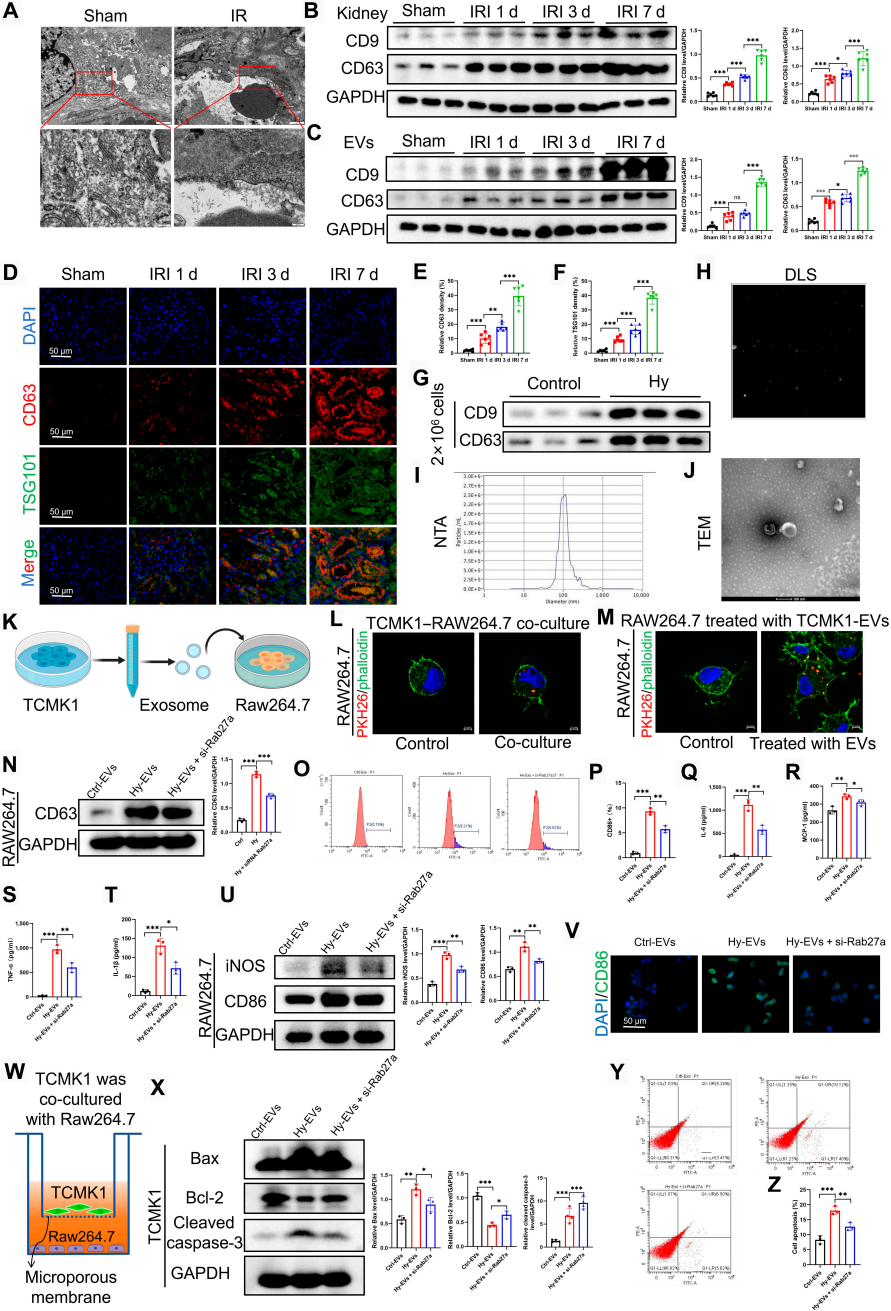
Tubular-cell-derived extracellular vesicles (EVs) promote macrophage M1 polarization and aggravate renal inflammation and injury. (A) Transmission electron microscopy (TEM) images showing extracellular vesicles in the kidneys of sham and ischemia-reperfusion injury (IRI) mice. (B and C) Western blot analysis of CD63 levels in EVs and kidney tissues at different time points post-IRI (*n* = 6). (D to F) Immunofluorescence and quantification of EVs’ markers CD63 and tumor susceptibility gene 101 (TSG101) in renal tissues (*n* = 6). (G to J) Validation of EV identity from TCMK1 cells via Western blot, dynamic light scattering (DLS), nanoparticle tracking analysis (NTA), and TEM. (K to M) Schematic diagrams and images showing uptake of labeled EVs by Raw264.7 macrophages in direct and co-culture systems. (N) EV uptake assessed by CD63 expression in Raw264.7 cells treated with EVs from normoxic, hypoxia/reoxygenation (H/R)-treated, or Rab27a-silenced TCMK1 cells (*n* = 3). (O and P) Flow cytometry for CD86 in macrophages from different groups (*n* = 3). (Q to T) Enzyme-linked immunosorbent assay (ELISA) analysis of inflammatory cytokines (interleukin-6 [IL-6], monocyte chemoattractant protein-1 [MCP-1], tumor necrosis factor-α [TNF-α], and interleukin-1β [IL-1β]) in macrophage supernatants (*n* = 3). (U) Western blot of inducible nitric oxide synthase (iNOS) and CD86 in macrophages (*n* = 3). (V) Representative CD86 immunofluorescence images. (W) Diagram of the co-culture setup with TCMK1 and Raw264.7 cells under different conditions. (X) Western blot analysis of apoptosis-related proteins in TCMK1 cells. (Y and Z) Flow cytometry of apoptosis in TCMK1 cells co-cultured with differently treated macrophages (*n* = 3). Inhibiting EVs’ secretion by Rab27a knockdown reduced M1 polarization, cytokine production, and tubular epithelial cell (TEC) apoptosis. *P < 0.05; **P < 0.01; ***P < 0.001. IR, ischemia–reperfusion; GAPDH, glyceraldehyde-3-phosphate dehydrogenase; DAPI, 4′,6-diamidino-2-phenylindole; Hy-EVs, H/R-treated TCMK1 cell-derived EVs; FITC, fluorescein isothiocyanate; Bax, Bcl-2-associated X protein; Bcl-2, B-cell lymphoma 2; PE, phycoerythrin.

The isolated EVs measured around 50 nm in diameter, and we therefore propose that EV-mediated communication contributes to macrophage inflammatory responses. Transmission electron microscopy (TEM) showed vesicles (30 to 150 nm) in kidneys 1 d post-IRI (Fig. 1A), with CD9 and CD63 (tetraspanin family proteins enriched on the membrane of EVs and commonly used as EV markers) increasing over the week (Fig. 1B and C). EVs from kidney tissue contained impurities, so glyceraldehyde-3-phosphate dehydrogenase was used as an internal reference [23]. Immunofluorescence staining of CD63 and tumor susceptibility gene 101 (TSG101, a component of the endosomal sorting complex required for transport complex involved in EV biogenesis) further confirmed these findings (Fig. 1D to F) [24].

In vitro, TCMK1 cells were exposed to normoxia or hypoxia/reoxygenation (H/R) conditions. Western blot confirmed CD9 and CD63 (EVs’ markers) (Fig. 1G). EV isolation from H/R-treated TCMK1 cells was verified by dynamic light scattering, nanoparticle tracking analysis, and TEM, showing a typical 30- to 150-nm cuplike shape (Fig. 1H to J).

Raw264.7 cells were exposed to Hy-EVs (H/R-treated TCMK1 cell-derived EVs) (Fig. 1K). EV uptake was tracked by PKH26 labeling and phalloidin staining (Fig. 1M). A co-culture system confirmed EV internalization, with Raw264.7 cells in the bottom chamber and TCMK1 cells at the top, separated by a microporous barrier (Fig. 1L).

Rab27a, a small GTPase, facilitates EVs’ release by aiding multivesicular body fusion with the membrane [25]. Rab27a recruits Slp2a and Munc13-4 to aid multivesicular body movement and fusion with the plasma membrane, regulating EVs’ release efficiency [26–28]. Inhibiting Rab27a (e.g., via small interfering RNA [siRNA]) reduced EVs’ secretion [29,30]. Rab27a is crucial for EVs’ secretion, but its role in other secretion pathways, such as cytokine release, appears minimal. Knockout (KO) of Rab27a reduces EVs’ secretion without substantially affecting cytokine or protein release, suggesting its specific function in the EVs’ pathway [31,32]. TCMK1 cells were transfected with si-Rab27a to reduce EVs’ secretion. EV uptake by Raw264.7 macrophages decreased, evidenced by lower CD63 levels (Fig. 1N). Flow cytometry and Western blotting showed that EVs from hypoxia-treated TCMK1 cells promoted M1 macrophage polarization (Fig. 1O, P, and U).

Pro-inflammatory cytokine levels (IL-1β, IL-6, TNF-α, and monocyte chemoattractant protein-1) in macrophage media were measured by enzyme-linked immunosorbent assay (ELISA) (Fig. 1Q to T) and mRNA (Fig. S4A to D). Immunofluorescence showed that EVs from renal tubular cells induced M1 polarization, and Rab27a inhibition reduced it (Fig. 1V and Fig. S4E). Detection of the mRNA levels of macrophage polarization markers also confirmed the promotion of macrophage polarization (mainly M1) by EVs (Fig. S4F to I). Co-culture experiments showed that M1 macrophages enhanced TCMK1 cell apoptosis, as confirmed by flow cytometry (Fig. 1W to Z).

**Fig. 2.**
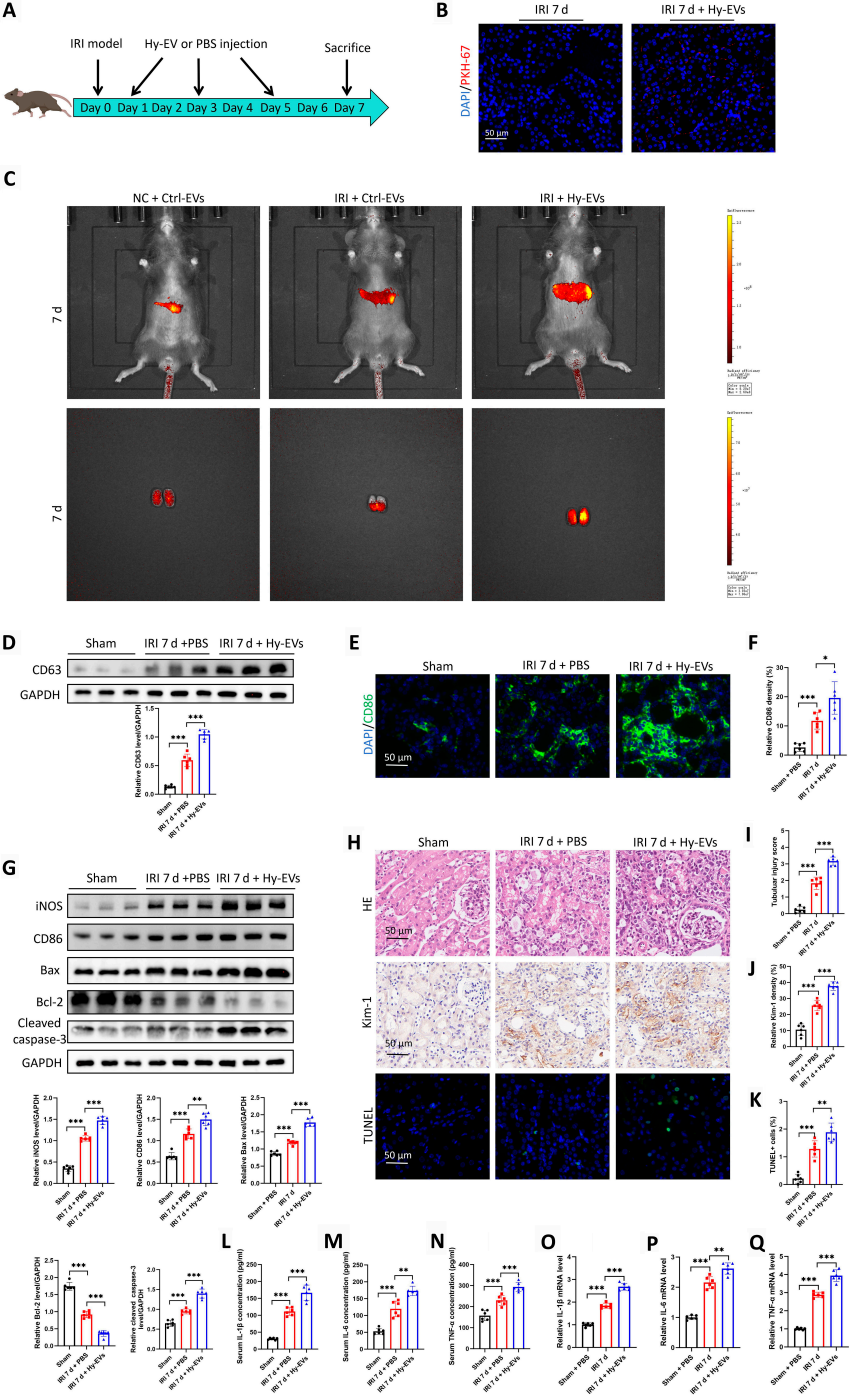
In vivo, tubule-derived EVs worsen renal damage, increase inflammation, and encourage macrophage M1 polarization. (A) The experimental design is shown schematically. EVs from H/R-treated TCMK1 cells (Hy-EVs) were injected via the tail vein on days 1, 3, and 5 after surgery in a mouse IRI model. On the seventh day, the kidney tissues were taken. (B) PKH-67-labeled Hy-EV visualization in mouse kidney tissues after injection into the tail vein. PKH-67-labeled EVs (red) are shown. (C) In vivo imaging was performed to track 1,1′-dioctadecyl-3,3,3′,3′-tetramethylindotricarbocyanine iodide (DiR)-labeled EVs and evaluate EV uptake efficiency by the kidneys after IRI modeling. (D) CD63 expression in the tissues of the kidneys from IRI mice given either phosphate-buffered saline (PBS) or Hy-EV injections (*n* = 6) was examined using Western blot. (E and F) Illustrative CD86 immunofluorescence pictures in kidney tissues from IRI mice given either PBS or Hy-EV injections. (G) Western blot examination of Bax, Bcl-2, cleaved caspase-3, iNOS, and CD86 in kidney tissues from IRI mice given either PBS or Hy-EV injections (*n* = 6). (H) Typical kidney histopathology pictures as determined by hematoxylin and eosin (HE) staining. (I) Kidney injury molecule-1 (Kim-1) immunohistochemical staining in the tissues of the kidneys from IRI mice given Hy-EV or PBS injections. (J) Kidney tissues from IRI mice given PBS or Hy-EV injections are shown with representative terminal deoxynucleotidyl transferase dUTP nick-end labeling (TUNEL) staining. (K to N) ELISA was used to measure the serum levels of pro-inflammatory cytokines (IL-6, IL-1β, TNF-α, and MCP-1) in several mouse groups. (O to Q) Polymerase chain reaction (PCR) was used to determine the messenger RNA (mRNA) levels of renal inflammatory factors in kidney tissues from IRI mice that received either PBS or Hy-EV injections (*n* = 6). *P < 0.05; **P < 0.01; ***P < 0.001. NC, negative control.

A renal IRI mouse model was generated, and Hy-EVs (from hypoxia-treated TCMK1 cells) were injected on days 1, 3, and 5. On day 7, kidney tissues were collected. PKH-67-labeled Hy-EVs were tracked and visualized in kidney tissues (Fig. 2A and B). To further investigate whether intravenously administered EVs can reach the kidney, in vivo fluorescence imaging was conducted in mice. 1,1′-Dioctadecyl-3,3,3′,3′-tetramethylindotricarbocyanine iodide-labeled control TCMK1 cells (Ctrl-Exo) or Hy-EVs were injected via the tail vein on days 1, 3, and 5 after IRI. The biodistribution of EVs was monitored by imaging the lungs, liver, and kidneys on days 1, 3, 5, and 7. For mice that received EVs on the same day, imaging was performed 6 h after injection. Fluorescence intensity increased progressively over time, with the lungs showing the highest accumulation, followed by the liver. The kidney consistently exhibited relatively low fluorescence signals at all time points (Fig. S5A and Fig. 2C). After adjusting for background fluorescence from other organs, a gradual increase in the kidney-associated signal was observed, peaking on day 7 (Fig. S5B). Our fluorescence in situ hybridization analysis confirmed that EVs derived from H/R-treated TECs transfer miR-491-3p into kidney parenchymal cells following IRI, leading to its substantially accumulation within renal tissue (Fig. S6).

CD63 protein levels were measured in sham + phosphate-buffered saline, IRI 7 d, and IRI 7 d + Hy-EVs groups. Western blot showed higher CD63 expression in the IRI 7 d + Hy-EVs group (Fig. 2D). Immunofluorescence confirmed Hy-EV treatment enhanced M1 macrophage polarization in the kidney (Fig. 2E and F). M1 markers CD86 and iNOS were elevated in the IRI 7 d + Hy-EVs group, confirming Hy-EVs’s role in M1 polarization (Fig. 2G). mRNA and serum ELISA showed that Hy-EVs exacerbated inflammation via M1 polarization (Fig. 2L to Q).

Western blotting showed elevated pro-apoptotic proteins Bax (Bcl-2-associated X protein), Bcl-2 (B-cell lymphoma 2), and cleaved caspase-3 in Hy-EV-treated kidney tissues (Fig. 2G). Hematoxylin and eosin staining, Kim-1 immunohistochemistry, and TUNEL staining confirmed exacerbated renal damage and increased apoptosis in the Hy-EVs group (Fig. 2H to K). These results reflect the effects of exogenously administered Hy-EVs on macrophage polarization and renal IRI, but we will next focus on inhibiting endogenous EVs’ secretion to investigate further.

### In vivo, inhibiting EVs’ secretion reduces macrophage M1 polarization to alleviate renal IRI

Rab27a KO mice were generated and confirmed by gel electrophoresis (Fig. S7A and B). PCR analysis revealed that Rab27a mRNA expression in KO kidneys was down-regulated by several-hundred-fold compared to that in wild-type (WT) controls, indicating effective gene deletion (Fig. S7C). Western blotting showed reduced EVs’ secretion and CD63 expression in Rab27a KO mice (Fig. S7D). M1 polarization markers CD86 and iNOS were lower, indicating attenuated macrophage polarization (Fig. S7I). Immunofluorescence confirmed reduced M1 macrophages in KO mice despite unchanged macrophage infiltration (Fig. S7J to M).

Rab27a KO reduced renal injury, tubular expansion, and apoptosis. Immunohistochemistry and Western blot showed lower Kim-1 and apoptotic markers (Bax and cleaved caspase-3) in KO mice (Fig. S7E to I).

PCR and ELISA showed reduced IL-1β, IL-6, and TNF-α levels in Rab27a KO mice, indicating that Rab27a KO alleviates renal IRI by inhibiting EVs’ release, M1 macrophage polarization, and renal inflammation and apoptosis (Fig. S7N to S).

### EV-carried miR-491-3p from tubular cells enhances macrophage inflammatory polarization in vitro

**Fig. 3.**
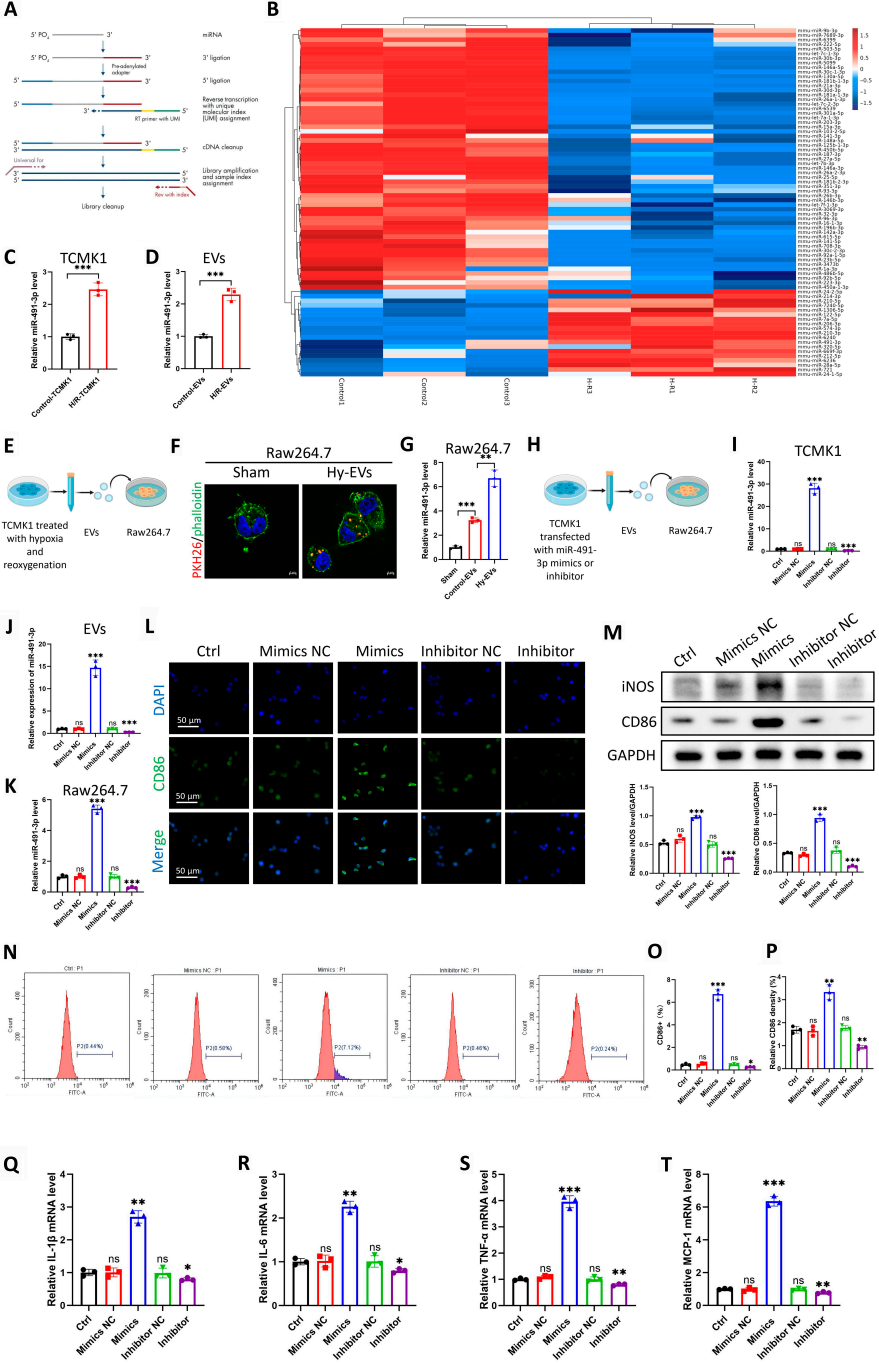
EV-carried miR-491-3p produced from tubular cells stimulates the inflammatory response and macrophage M1 polarization in vitro. (A) EV-carried microRNA (miRNA) sequencing workflow. (B) Heatmap of TCMK1 cells’ EV-carried miRNA expression levels after H/R therapy. (C) PCR examination of TCMK1 cells’ expression of miR-491-3p after H/R treatment (*n* = 3). (D) PCR examination of miR-491-3p expression in TCMK1 cell EVs after H/R treatment (*n* = 3). (E) The experimental setup’s schematic design. Raw264.7 cells were treated with EVs obtained from TCMK1 cells treated with H/R. (F) TCMK1 cell EVs tagged with PKH26 and Raw264.7 cell membranes stained with phalloidin demonstrating EV uptake. (G) PCR study of the expression of miR-491-3p in Raw264.7 cells treated with EVs from TCMK1 cells treated with H/R (*n* = 3). (H) The experimental setup’s schematic design. Inhibitors or mimics of miR-491-3p were transfected into TCMK1 cells. Following H/R therapy, EVs were extracted and used to treat Raw264.7 cells. (I) PCR study of the expression of miR-491-3p in TCMK1 cells transfected with inhibitors or mimics of miR-491-3p (*n* = 3). (J) miR-491-3p expression in EVs from TCMK1 cells transfected with inhibitors or mimics of miR-491-3p (*n* = 3) was examined by PCR. (K) PCR study of the expression of miR-491-3p in Raw264.7 cells treated with EVs from TCMK1 cells (*n* = 3) transfected with miR-491-3p mimics/inhibitors. (L and P) In Raw264.7 cells treated with EVs from various groups, M1 polarization is visible by immunofluorescence labeling of CD86. (M) iNOS and CD86 protein levels in Raw264.7 cells from various treatment groups (*n* = 3) were analyzed using Western blot. (N and O) Flow cytometry study of Raw264.7 cells from 3 distinct groups using the M1 polarization marker CD86. (Q to T) The expression of inflammatory cytokines from various groups (*n* = 3) was examined using PCR. ns, not significant; *P < 0.05; **P < 0.01; ***P < 0.001. cDNA, complementary DNA.

Tubular-cell-derived EVs regulate macrophage function in IRI, but the mechanisms remain unclear. EVs deliver microRNAs (miRNAs) that regulate gene expression in target cells [19,33]. EVs from H/R-treated TCMK1 cells were isolated for miRNA sequencing (Fig. 3A). Using |Log FC| > 0.5 and *P* < 0.05, 12 up-regulated miRNAs were identified, including 5 mouse-specific and 7 conserved miRNAs (Fig. 3B and Fig. S8A). After excluding miRNAs with false discovery rate > 0.1, 4 biologically significant miRNAs—miR-491-3p, miR-214-3p, miR-210-3p, and miR-24-2-5p—were selected. Their expression levels were measured in both TCMK1 cells and EVs (Fig. S8C to J), with mature sequences showing high conservation between humans and mice (Fig. S8K).

Kyoto Encyclopedia of Genes and Genomes (KEGG) pathway enrichment analysis via DIANA-miRPath revealed that miR-491-3p was significantly associated with NF-κB signaling, lysine metabolism, apoptosis, and ubiquitin-dependent degradation. Notably, the NF-κB pathway, a key regulator of inflammation, highlighted miR-491-3p as a potential mediator in inflammation. The other 3 miRNAs were mainly enriched in tumor-related pathways (Fig. S9).

Raw264.7 macrophages were transfected with mimics of miR-491-3p, miR-214-3p, miR-210-3p, and miR-24-2-5p, with transfection efficiency confirmed by PCR (Fig. S10A to D). Only miR-491-3p significantly enhanced M1 polarization, shown by increased CD86 and iNOS expression (Fig. S10E to H) and confirmed by immunofluorescence (Fig. S11). miR-491-3p levels in renal tissue and EVs were measured at 1, 3, and 7 d after IRI, showing a sharp increase that remained elevated for at least 1 week (Fig. S12). These findings indicate that miR-491-3p, released from renal TECs, plays a key role in M1 polarization, inflammation, and renal damage during IRI.

PCR showed increased miR-491-3p in TCMK1 cells and EVs (Fig. 3C and D). Exposure to Hy-EVs elevated miR-491-3p in Raw264.7 cells (Fig. 3E to G). miR-491-3p mimics or inhibitors were transfected into TCMK1 cells, and EVs were used to treat Raw264.7 macrophages (Fig. 3H and I). The transfer of EV-carried miR-491-3p to macrophages was efficient, maintaining elevated expression in Raw264.7 cells (Fig. 3J and K). miR-491-3p was efficiently transferred, increasing M1 markers (iNOS and CD86) and shifting macrophages to the M1 phenotype while inhibiting M2 polarization (Fig. 3L to P and Fig. S13A to D). Cytokine release was elevated (Fig. 3Q to T). Inhibition of miR-491-3p reduced M1 markers and cytokine secretion, confirming its role in inflammation.

Our additional experiments using human HK-2 and THP-1 cells confirmed that EVs derived from TECs treated with H/R transfer miR-491-3p to macrophages, inducing M1 polarization and pro-inflammatory cytokine expression. Inhibition of miR-491-3p attenuated these effects. These findings support that the mechanisms identified in murine models are conserved in human systems, reinforcing the translational significance of TEC-derived EV miR-491-3p in renal IRI (Fig. S14).

The results highlight that EVs’ transfer of miR-491-3p is crucial for H/R-induced inflammation, promoting macrophage M1 polarization and increasing pro-inflammatory cytokine production.

### EV-carried miR-491-3p from renal tubules enhances macrophage-mediated renal inflammation in IRI in vivo

**Fig. 4.**
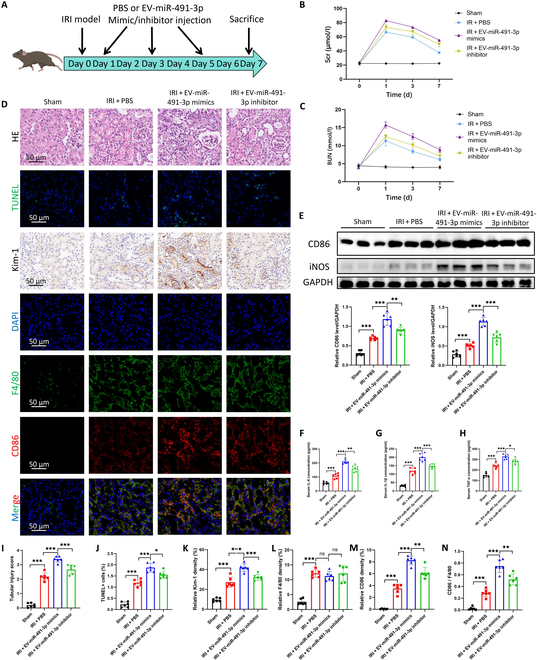
EV-carried miR-491-3p modulates renal IRI in vivo. (A) Schematic of the experimental design for the renal IRI mouse model. EVs derived from hypoxia-treated TCMK1 cells transfected with miR-491-3p mimics or inhibitors were injected on days 1, 3, and 5 after IRI induction, and kidney samples were collected on day 7 for analysis. (B and C) Serum creatinine (Scr) and blood urea nitrogen (BUN) levels. (D) Representative images of renal histological and immunofluorescence staining. HE staining shows structural damage in kidney tissues. TUNEL staining indicates apoptotic cells. Immunohistochemistry for Kim-1 highlights tubular injury. Double immunofluorescence staining for F4/80 (green) and CD86 (red) reveals macrophage infiltration and M1 polarization, with nuclear counterstaining by DAPI (blue). (E) Western blot analysis of CD86, iNOS, and GAPDH protein levels in kidney tissues. Relative protein levels of CD86 and iNOS are quantified in the bar graph (*n* = 6). (F to H) ELISA results showing the serum concentrations of IL-1β, IL-6, and TNF-α in different groups. The levels of these inflammatory cytokines were significantly elevated in the IRI + Exo-miR-491-3p mimics group and reduced in the inhibitor group (*n* = 6). (I to N) Quantitative analysis of renal injury markers: tubular injury score (I), tubular dilation percentage (J), Kim-1 (K), F4/80 (L), and CD86 (M and N) densities. *P < 0.05; **P < 0.01; ***P < 0.001.

An IRI mouse model was established to study the effects of EV-carried miR-491-3p on renal IRI. EVs from hypoxia-treated TCMK1 cells transfected with miR-491-3p mimics or inhibitors were administered on days 1, 3, and 5 post-IRI, with kidney samples collected on day 7 (Fig. 4A). Serum creatinine and blood urea nitrogen levels were measured at 36 h, 88 h, and 7 d post-IRI. miR-491-3p mimics worsened renal dysfunction, while the inhibitors alleviated kidney damage (Fig. 4B and C). Histological analysis showed that EV-carried miR-491-3p worsened renal injury, apoptosis, and M1 macrophage polarization without changing infiltration (Fig. 4D and I to M). Western blot and quantitative polymerase chain reaction (qPCR) confirmed elevated M1 markers (CD86 and iNOS), and cytokines (IL-1β, IL-6, and TNF-α) were increased in the kidney and serum (Fig. 4F to H and Fig. S15A to E). In contrast, miR-491-3p-enriched EVs inhibited macrophage M2 polarization (Fig. S15F and G). These findings suggest that miR-491-3p promotes M1 polarization, exacerbating renal inflammation and damage in vivo.

### Renal TEC-derived EV-carried miR-491-3p directly targets SIRT1 and substantially inhibits its transcription and protein expression in macrophages

**Fig. 5.**
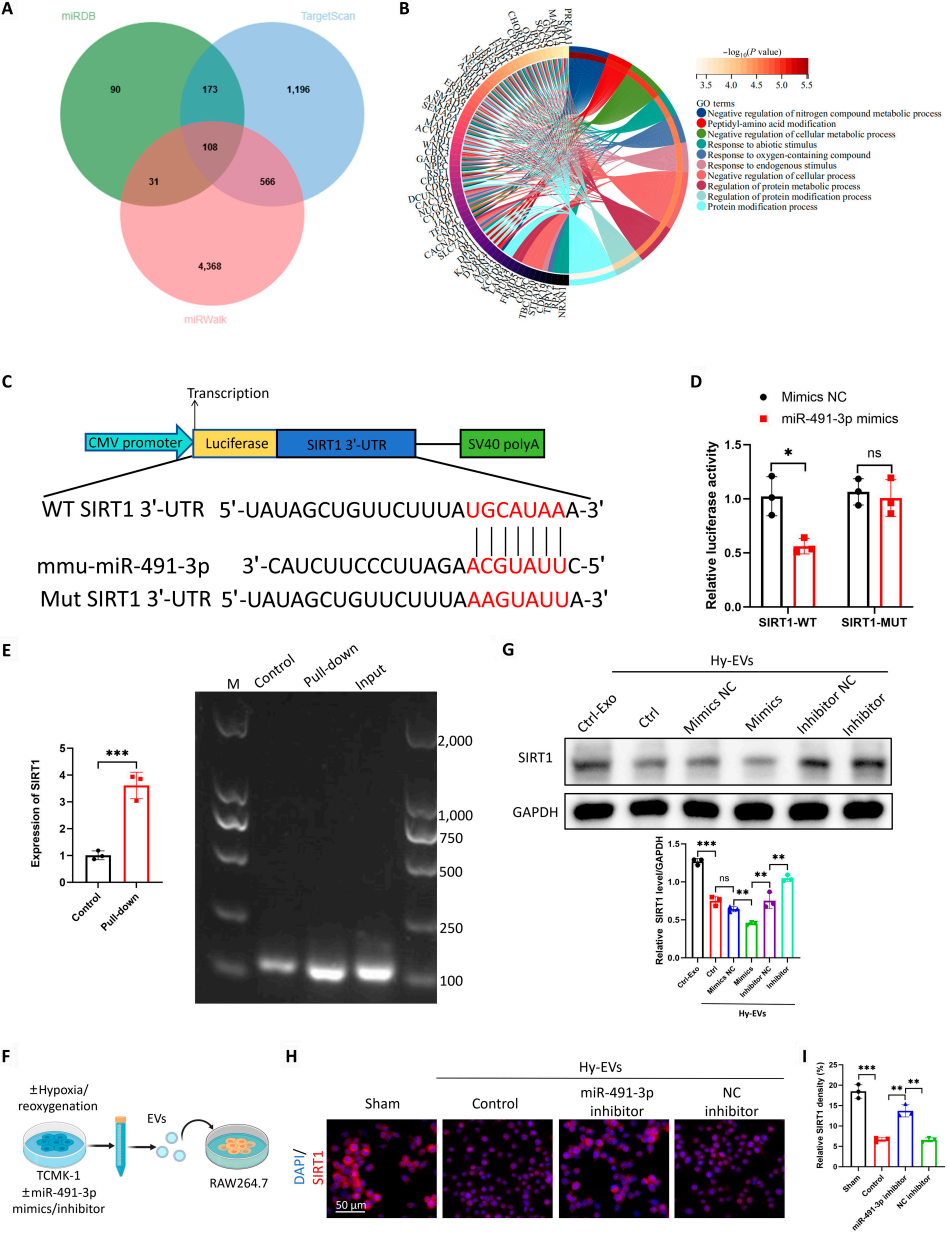
EV-carried miR-491-3p generated from tubular cells directly targets sirtuin 1 (SIRT1). (A) Using the miRDB, TargetScan, and miRWalk databases, miR-491-3p target gene predictions are made. A total of 108 target genes that intersected were found. (B) The 108 target genes were subjected to Gene Ontology (GO) enrichment analysis, which highlighted functional categories associated with metabolic control and protein modification. (C) TargetScan-identified miR-491-3p predicted binding location in the 3′ untranslated region (3′-UTR) of SIRT1 mRNA. (D) Luciferase activity test in Raw264.7 cells co-transfected with mouse luciferase reporter vectors carrying the wild-type or mutant 3′-UTR of SIRT1 or NC or miR-491-3p mimic. (E) RNA pull-down test in Raw264.7 macrophages using a miR-491-3p probe tagged with biotin. Quantitative polymerase chain reaction (qPCR) was used to identify SIRT1 mRNA bound to miR-491-3p, and agarose gel electrophoresis was used to corroborate the results. (F) Experimental design schematic diagram. EVs were isolated and used to treat Raw264.7 cells after TCMK1 cells were transfected with miR-491-3p mimics or inhibitors and treated with H/R. SIRT1 protein expression in Raw264.7 cells treated with EVs from hypoxia-treated TCMK1 cells (Hy-EVs), control TCMK1 cells (Ctrl-Exo), Hy-EVs + miR-491-3p mimics, and Hy-EVs + miR-491-3p inhibitors (*n* = 3) is shown in (G) Western blot analysis. (H) Illustrative immunofluorescence pictures demonstrating SIRT1 expression in Raw264.7 cells from several groups treated with EVs. (I) SIRT1 fluorescence intensity in Raw264.7 cells (*n* = 3) was quantitatively analyzed. *P < 0.05; **P < 0.01; ***P < 0.001. WT, wild type.

Using miRDB, TargetScan, and miRWalk, 108 potential miR-491-3p target genes were identified (Fig. 5A), mostly related to metabolic regulation and protein modification (Fig. 5B). Both TargetScan and miRDB scores rank sirtuin 1 (SIRT1) high, making it a focus for further study.

TargetScan identified conserved miR-491-3p binding sites in SIRT1’s 3′ untranslated region (Fig. 5C). Dual-luciferase reporter assays showed that miR-491-3p mimics reduced luciferase activity in Raw264.7 cells co-transfected with SIRT1-WT 3′ untranslated region plasmid, confirming direct binding (Fig. 5C and D). RNA pull-down assays further validated this interaction (Fig. 5E).

EVs from hypoxia-treated TCMK1 cells transfected with miR-491-3p mimics or inhibitors were used to assess their effect on SIRT1 expression in Raw264.7 cells. Western blotting showed reduced SIRT1 levels in cells treated with EV-carried miR-491-3p (Fig. 5F and G). Immunofluorescence confirmed this reduction, with the miR-491-3p inhibitor partially restoring SIRT1 expression (Fig. 5H and I). These results indicate that EV-carried miR-491-3p targets SIRT1, decreasing its expression and promoting macrophage M1 polarization.

### SIRT1 regulates macrophage M1 polarization

Single-cell RNA sequencing data from human kidney tissues were obtained from an open-access database (https://atlas.kpmp.org/explorer/). The single-cell RNA sequencing revealed a significant decrease in SIRT1 mRNA expression in renal macrophages from acute kidney injury (AKI) or CKD patients (Fig. S16A), suggesting its involvement in macrophage inflammation. Gene Ontology enrichment analysis of SIRT1 via the Enrichr database identified pathways related to inflammation, including toll-like receptor and interferon-gamma signaling (Fig. S16B).

SIRT1 was overexpressed in RAW264.7 macrophages, confirmed by PCR (Fig. S16C). Lipopolysaccharide-induced M1 polarization was partially reversed by SIRT1 overexpression, reducing M1 markers and CD86 expression (Fig. S16D and E). These findings suggest that SIRT1 inhibits M1 macrophage polarization and mitigates inflammation.

### EV-carried miR-491-3p promotes macrophage M1 polarization via SIRT1-mediated deacetylation of NICD to activate the Notch/NF-κB signaling pathway in vitro

**Fig. 6.**
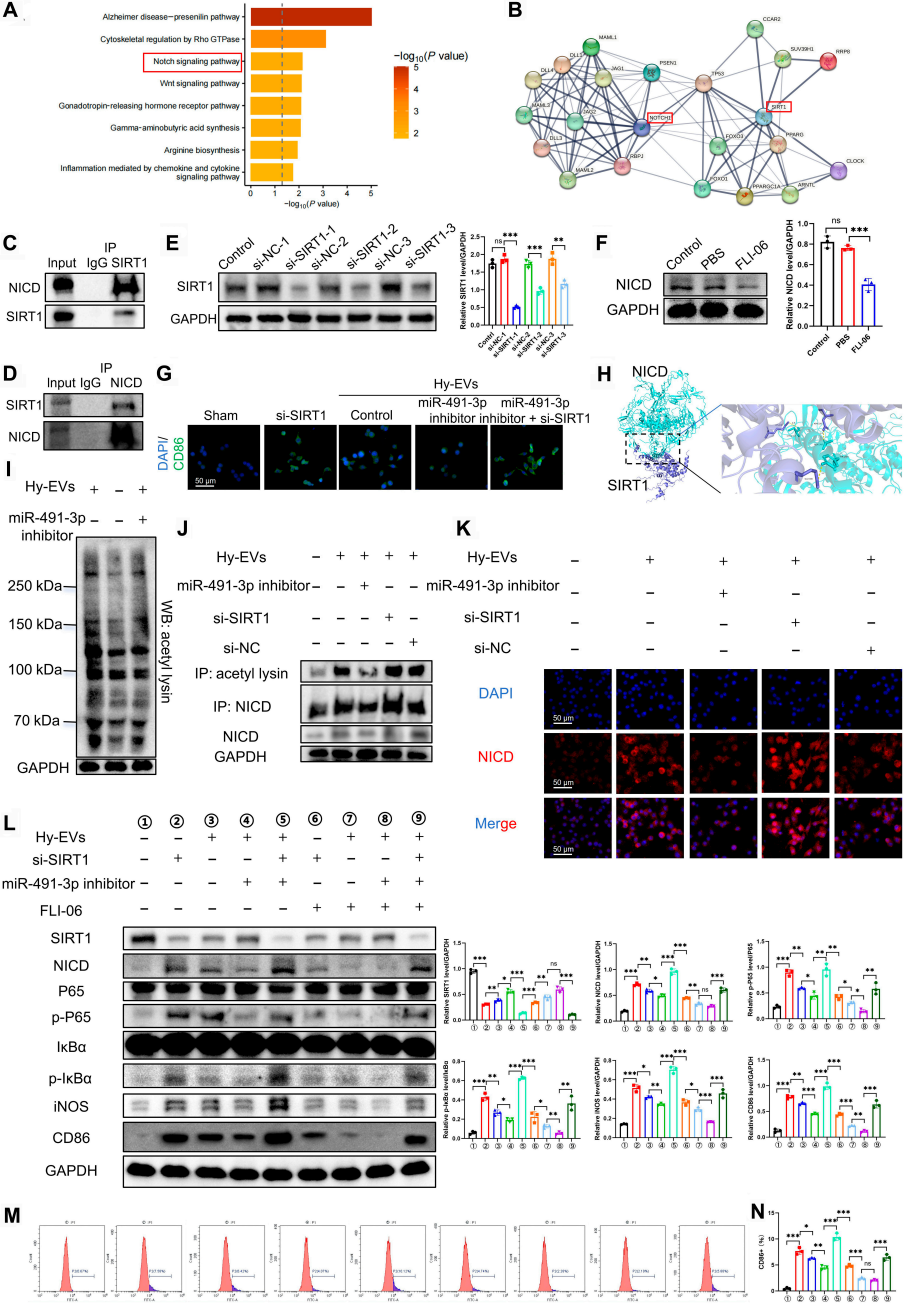
EV-carried miR-491-3p produced from TECs inhibits SIRT1-mediated deacetylation of Notch intracellular domain (NICD), stimulates the Notch/NF-κB signaling pathway, and facilitates M1 macrophage polarization. (A) Kyoto Encyclopedia of Genes and Genomes (KEGG) pathway analysis revealed that SIRT1 is significantly associated with the Notch signaling pathway (top 10 pathways shown). (B) Protein–protein interaction (PPI) network of SIRT1, showing its interaction with Notch1. (C and D) Co-immunoprecipitation (Co-IP) and Western blot analysis confirmed the interaction between SIRT1 and NICD in Raw264.7 cells. (E) Confirming SIRT1 knockdown efficiency in using si-SIRT1 (*n* = 3). (F) Confirming inhibition of the Notch pathway by FLI-06 (*n* = 3). (G) Immunofluorescence staining of CD86. (H) Structural analysis of SIRT1 and NICD showing peptide chain conformation and interacting binding sites. (I) Western blot analysis of global protein acetylation in Raw264.7 cells treated with Hy-EVs or Hy-EV-miR-491-3p inhibitor (*n* = 3). (J) Co-IP assay to assess NICD acetylation in Raw264.7 cells treated with Hy-EVs, Hy-EV-miR-491-3p inhibitor, Hy-EVs + si-SIRT1, or Hy-EVs + si-NC. (K) Immunofluorescence analysis of NICD fluorescence intensity in Raw264.7 cells treated with si-SIRT1, Hy-EVs, Hy-EV-miR-491-3p inhibitor, or Hy-EV-miR-491-3p inhibitor + si-SIRT1. (L) Western blot analysis of SIRT1; NICD; P65; p-P65; inhibitor of nuclear factor kappa B, alpha (IκBα); p-IκBα; iNOS; and CD86 in Raw264.7 cells treated with si-SIRT1, Hy-EVs, Hy-EV-miR-491-3p inhibitor, Hy-EV-miR-491-3p inhibitor + si-SIRT1, si-SIRT1 + FLI-06, Hy-EV + FLI-06, Hy-EV-miR-491-3p inhibitor + FLI-06, and Hy-EV-miR-491-3p inhibitor + si-SIRT1 + FLI-06. (M and N) Flow cytometry analysis of M1 polarization in Raw264.7 cells from the indicated treatment groups. *P < 0.05; **P < 0.01; ***P < 0.001. IgG, immunoglobulin G; WB, Western blot.

Pathway enrichment analysis revealed a strong link between SIRT1 and the Notch signaling pathway (Fig. 6A). Given the Notch pathway’s role in inflammation regulation, SIRT1 may modulate macrophage polarization through this signaling route [34,35]. Notch1 silencing in Raw264.7 cells via siRNA reduced lipopolysaccharide-induced M1 polarization markers, indicating that Notch1 signaling positively regulates macrophage M1 polarization and promotes a pro-inflammatory phenotype (Fig. S17).

Protein–protein interaction network analysis revealed interactions between SIRT1 and Notch1 (Fig. 6B). Notch intracellular domain (NICD), the active fragment of Notch signaling, is commonly used to assess Notch activation and its functional status [36]. Co-immunoprecipitation (Co-IP) assays confirmed SIRT1–NICD interaction (Fig. 6C and D). The knockdown effect of si-SIRT1 on SIRT1 and the inhibition effect of FLI-08 on Notch pathway were verified (Fig. 6E and F). Si-SIRT1 and Notch inhibitor FLI-06 enhanced CD86 fluorescence, indicating increased M1 polarization. Hy-EVs increased CD86, while miR-491-3p inhibitor reduced it; co-transfection with si-SIRT1 reversed this (Fig. 6G). Molecular docking showed the SIRT1–NICD binding conformation (Fig. 6H).

We hypothesized that EV-carried miR-491-3p influences NICD acetylation via SIRT1, modulating NICD pathway activity [37]. Acetylation, a key posttranslational modification, adds acetyl groups to lysine residues, altering protein properties and function via acetyltransferases [38]. Acetylation levels in different cell groups were analyzed using pan-acetylated lysine antibodies, showing a notable increase in the 100- to 150-kDa range after Hy-EV treatment, matching NICD’s molecular weight. The Hy-EV-miR-491-3p inhibitor group showed intermediate acetylation levels compared to the Hy-EVs and sham groups (Fig. 6I). Co-IP analysis further revealed that Hy-EVs enhanced NICD acetylation, which was reduced by miR-491-3p, while co-treatment with Hy-EVs and si-SIRT1 increased acetylation further (Fig. 6J). EV-carried miR-491-3p elevated NICD protein levels by inhibiting SIRT1-mediated deacetylation (Fig. 6K and L). Finally, Western blot and flow cytometry confirmed that tubule-derived EV-carried miR-491-3p activates the Notch/NF-κB pathway via SIRT1-mediated NICD deacetylation, promoting macrophage inflammatory polarization (Fig. 6L to N).

### MiR-491-3p prevents FBXW7-mediated ubiquitin-dependent degradation of NICD by inhibiting SIRT1-mediated NICD deacetylation

**Fig. 7.**
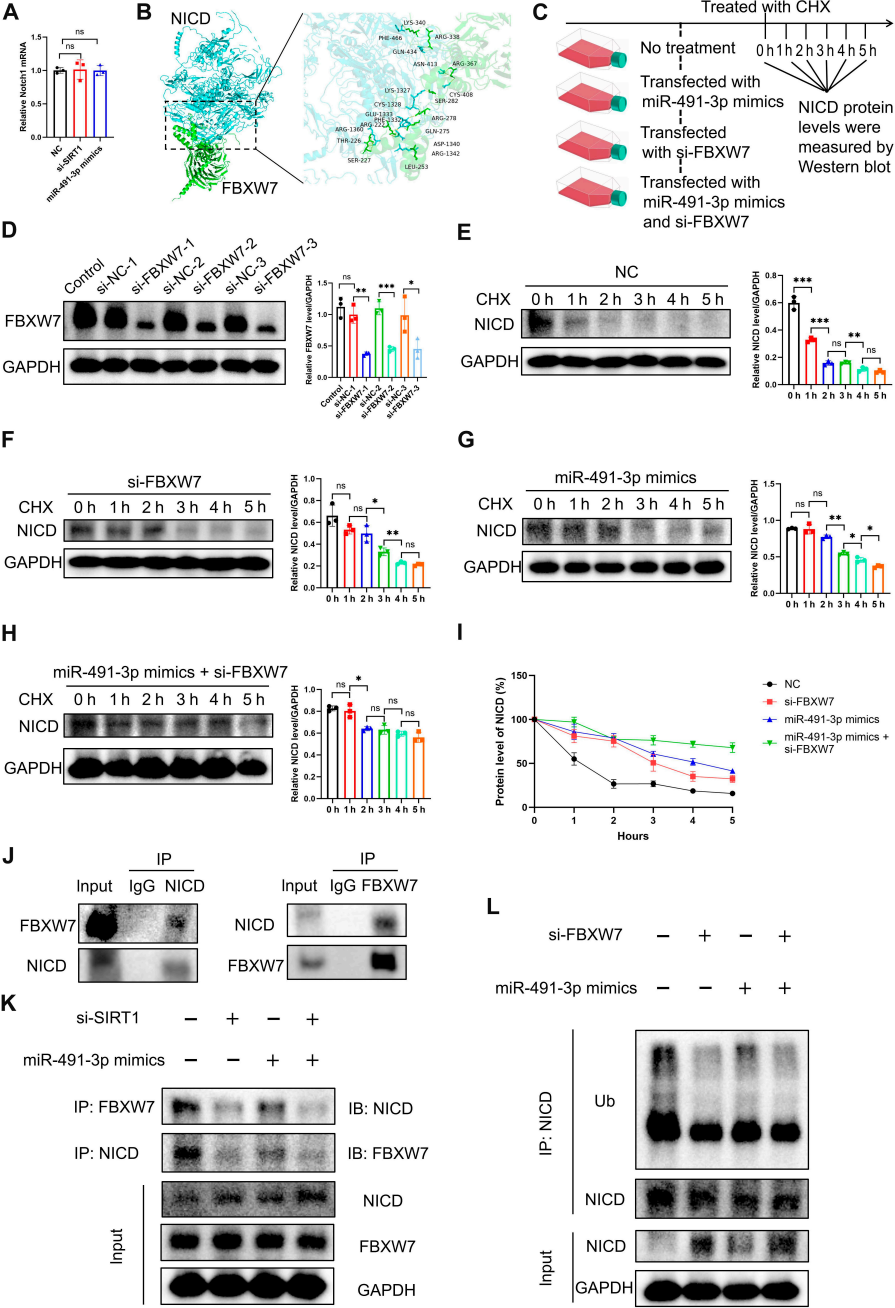
miR-491-3p modulates NICD acetylation via SIRT1, inhibits F-box and WD repeat domain-containing 7 (FBXW7)-mediated NICD ubiquitination, and impairs NICD degradation. (A) Notch1 mRNA levels were quantified by qPCR in the NC, si-SIRT1, and miR-491-3p mimics groups, showing no significant changes. (B) Molecular docking predicted the binding sites between FBXW7 and NICD. (C) Schematic representation of the experimental setup. (D) The knockdown efficiency of FBXW7 by si-FBXW7 in Raw264.7 cells was verified. (E to H) NICD protein levels were measured at different time points following cycloheximide (CHX) treatment in the NC, miR-491-3p mimics, si-FBXW7, and miR-491-3p mimics + si-FBXW7 groups. (I) NICD degradation rates were calculated based on protein levels detected in (E) to (H), indicating that miR-491-3p mimics and si-FBXW7 significantly slowed NICD degradation. (J) The Co-IP test validated the interaction involving FBXW7 and NICD in Raw264.7 cells. (K) Co-IP assays demonstrated that miR-491-3p mimics or si-SIRT1 reduced the interaction between FBXW7 and NICD in Raw264.7 cells. (L) Co-IP assays combined with MG132 treatment showed that miR-491-3p mimics reduced FBXW7-mediated ubiquitination of NICD. *P < 0.05; **P < 0.01; ***P < 0.001. Ub, ubiquitin.

The experiments suggest a positive correlation between acetylation levels and NICD protein expression. To explore whether acetylation affects transcription or protein degradation, we established 3 groups: negative control (NC), si-SIRT1, and miR-491-3p mimics. NICD mRNA expression is typically assessed through the Notch1 mRNA level [39]. PCR analysis showed no change in Notch1 mRNA (Fig. 7A), suggesting that NICD acetylation impacts its stability. F-box and WD repeat domain-containing 7 (FBXW7) mediates NICD ubiquitination and degradation via the 26S proteasome, regulating Notch signaling [40,41]. Molecular docking further predicted the binding sites between FBXW7 and NICD (Fig. 7B).

We investigated whether miR-491-3p regulates NICD degradation via FBXW7. Four groups were established: NC, miR-491-3p mimics, si-FBXW7, and miR-491-3p mimics + si-FBXW7. FBXW7 knockdown was confirmed by Western blot (Fig. 7C and D). Cells were treated with cycloheximide to block protein synthesis, and NICD degradation was monitored at different time points (Fig. 7E to H). We observed that both miR-491-3p mimics and si-FBXW7 significantly reduced the degradation rate of NICD (Fig. 7I).

Co-IP assays confirmed the interaction between FBXW7 and NICD (Fig. 7J). To explore if SIRT1 affects this interaction, we performed Co-IP assays and found that both miR-491-3p mimics and si-SIRT1 reduced the binding between FBXW7 and NICD (Fig. 7K). Further assays showed that miR-491-3p mimics decreased FBXW7-mediated NICD ubiquitination, which was enhanced by MG132 treatment (Fig. 7L).

In conclusion, miR-491-3p regulates NICD acetylation via SIRT1, impairing FBXW7 binding and inhibiting NICD degradation through the ubiquitin–proteasome pathway.

### In vivo, renal tubule-derived EV-carried miR-491-3p can activate the NF-κB pathway through the SIRT1/Notch axis to promote macrophage M1 polarization and renal IR-induced inflammatory response

**Fig. 8.**
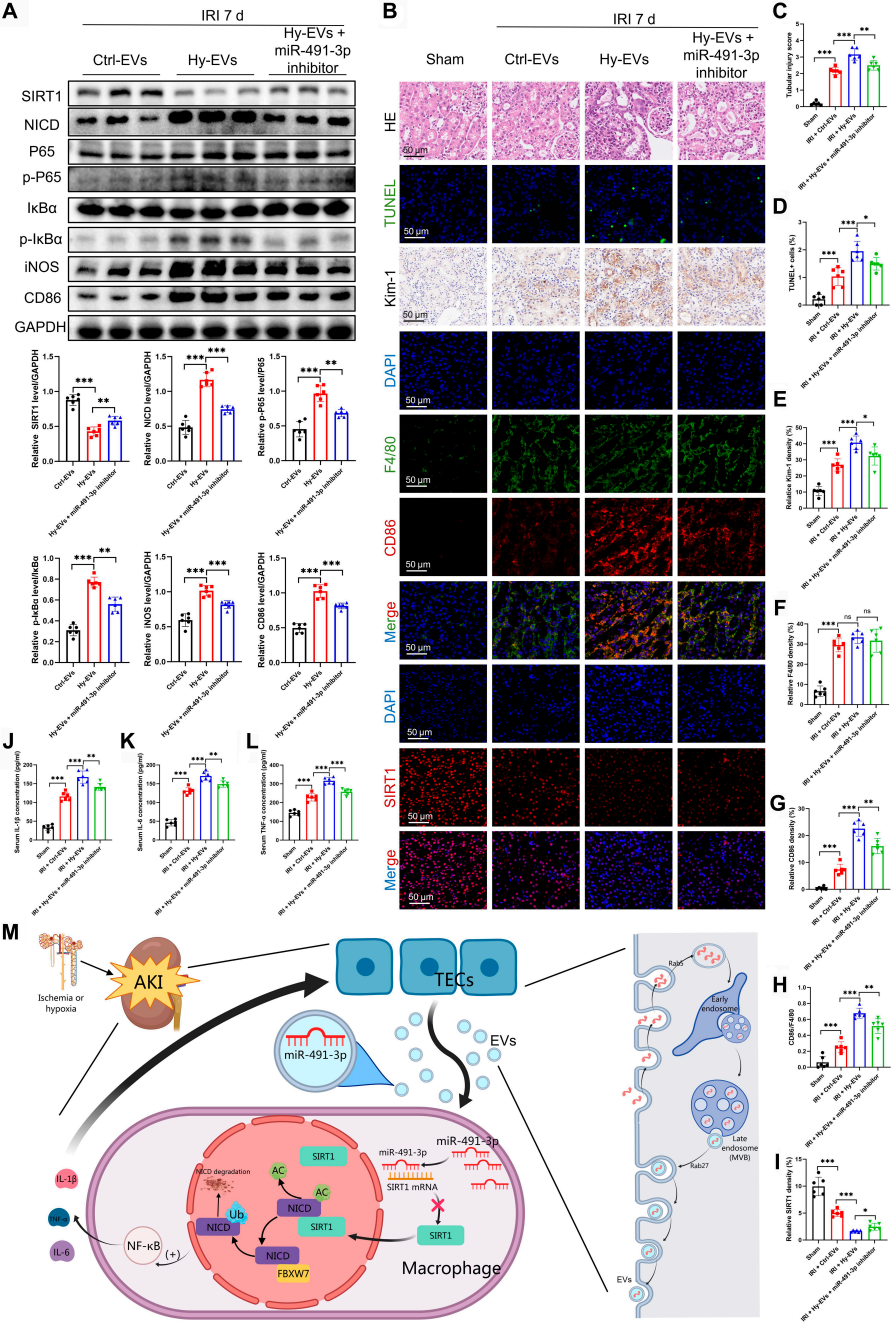
To confirm the mechanism by which EV-carried miR-491-3p produced from tubular cells regulates macrophage M1 polarization to exacerbate IR-induced kidney damage, in vivo tests were conducted. (A) Western blot analysis was used to determine the levels of SIRT1, NICD, P65, p-P65, IκBα, p-IκBα, iNOS, and CD86 in the kidneys of IRI 7 d mice given Ctrl-EVs, Hy-EVs, or Hy-EV-miR-491-3p inhibitor. (B to I) The kidneys of mice in various groups were stained with SIRT1 immunofluorescence, F4/80 and CD86 double immunofluorescence, HE staining, TUNEL staining, and Kim-1 immunohistochemistry. The photos and quantitative data are representative. (J to L) ELISA was used to measure the blood levels of TNF-α, IL-6, and IL-1β. (M) The graphic depicts how TEC-derived EV-carried miR-491-3p influences acute kidney injury (AKI) via the modulation of macrophage polarization, therefore intensifying renal inflammation and damage. In ischemia or hypoxic situations, TECs secrete EVs high in miR-491-3p, which are taken up by macrophages via a Rab27a-dependent secretion mechanism. Upon entering macrophages, miR-491-3p directly inhibits SIRT1 expression, hence compromising its function in deacetylating NICD. This defect hinders the interaction between NICD and the E3 ubiquitin ligase FBXW7, diminishing NICD ubiquitination and subsequent destruction. Stabilized NICD accumulates and activates the Notch and NF-κB signaling pathways, resulting in the production of pro-inflammatory cytokines including IL-1β, IL-6, and TNF-α. This directs macrophages into an M1-polarized inflammatory state, enhancing the inflammatory cascade and facilitating TEC apoptosis and renal tissue damage. Moreover, Rab27a loss may markedly inhibit EVs’ secretion, thereby diminishing macrophage M1 polarization and mitigating renal injury (*n* = 6). *P < 0.05; **P < 0.01; ***P < 0.001. AC, acetyl; MVB, multivesicular body.

In vivo, Hy-EV treatment reduced SIRT1, stabilized NICD, and activated the NF-κB pathway, evidenced by increased p-P65, p-IκBα, and M1 markers CD86 and iNOS (Fig. 8A). Histological analysis showed worsened kidney injury, with structural damage, apoptosis, and elevated Kim-1 (Fig. 8B to D). Immunofluorescence confirmed M1 macrophage infiltration (F4/80/CD86) and reduced SIRT1 (Fig. 8B and E to I). ELISA and PCR showed elevated pro-inflammatory cytokines in Hy-EV-treated kidneys, mitigated by miR-491-3p inhibition (Fig. 8J to L and Fig. S18). These results suggest that miR-491-3p in EVs from TECs inhibits SIRT1, impairing NICD deacetylation and promoting M1 polarization via NF-κB, exacerbating kidney injury. Blocking miR-491-3p or Rab27a reduced inflammation and kidney damage (Fig. 8M).

## Discussion

Renal IRI leads to marked damage of TECs due to hypoxia and high metabolic demand, followed by an inflammatory response upon reperfusion. We established a unilateral renal artery clamping model—retaining the contralateral kidney—to simulate localized IRI while reducing systemic burden and mortality. Compared with bilateral or contralateral nephrectomy models, this approach offers several distinct advantages: (a) it reduces perioperative mortality and better mimics clinical scenarios such as partial nephrectomy, renal artery embolization, or segmental infarction; (b) the intact contralateral kidney serves as an internal control, enabling clearer discrimination of localized versus systemic effects; (c) it permits longitudinal observation of the AKI-to-CKD transition in a survivable and clinically relevant context; and (d) it avoids confounding by uremia or volume overload often seen in bilateral IRI models, particularly when evaluating immune responses. These advantages support the use of the unilateral IRI model in our study to investigate local injury mechanisms and their systemic consequences [42]. Our findings identify EV-carried miR-491-3p, released from injured TECs, as a critical modulator of macrophage polarization. Mechanistically, miR-491-3p targets SIRT1, inhibits its deacetylation of NICD, and thereby stabilizes NICD by preventing its proteasomal degradation. The resulting activation of the Notch/NF-κB pathway promotes M1 macrophage polarization, exacerbating renal inflammation and tissue damage.

This study is the first to demonstrate that EV-carried miR-491-3p derived from hypoxic TECs directly regulates macrophage phenotype via the SIRT1/Notch axis. Our findings expand the understanding of intercellular communication in IRI and highlight TECs as active participants in shaping immune responses. We further confirm the central anti-inflammatory role of SIRT1 in macrophages, as its restoration reduces M1 polarization and renal injury. Moreover, using Rab27a KO mice, we show that inhibition of EVs’ secretion alleviates inflammatory damage, supporting the importance of EV-mediated signaling in kidney injury. These results establish a foundation for exploring EV-carried miRNAs as upstream regulators of immune cell behavior in renal disease.

Several limitations warrant consideration. First, while we confirmed miR-491-3p’s role in macrophage polarization, the in vivo specificity of its effect may be influenced by other immune or stromal cell populations not examined in this study. Second, the current model primarily captures acute-phase changes.

Recent studies have shown that EVs can serve not only as early diagnostic biomarkers for tumors but also as potential therapeutic targets [43]. Recent progress has highlighted the therapeutic promise of engineered cellular vesicles, including both natural EVs and synthetic EV mimics, for cancer and infectious disease immunotherapy [44,45]. This suggests that the TEC-derived EV-carried miR-491-3p identified in our study may likewise play an important role in the diagnosis and treatment of AKI. Given the pivotal role of EV-carried miR-491-3p in promoting inflammation, therapeutic strategies such as antagomiRs, siRNA-based silencing, or miRNA sponges may offer effective means to suppress its activity. Likewise, pharmacological activation of SIRT1 could counteract Notch pathway overactivation and mitigate renal injury.

However, translating these strategies to clinical application remains challenging. Current bottlenecks in AKI drug development include poor renal targeting, immune safety concerns, and inconsistent efficacy in human trials. We hold the view that a critical challenge in translating antagomiRs, siRNA, or SIRT1 activators into clinical therapies lies in effective renal delivery, as systemic administration often results in predominant uptake by the liver and lung, with limited accumulation in the kidney. Off-target effects and variable biodistribution remain major barriers for AKI drug development. Recent advances in EV engineering provide promising strategies to overcome these hurdles. Recent research studies have highlighted the need for precise delivery systems (e.g., kidney-targeting nanoparticles or engineered EVs) to overcome these hurdles [46–48]. For example, Tang et al. [49] demonstrated that decorating EVs with the Kim-1 binding peptide (LTH) enabled specific targeting of injured proximal tubules and efficient RNA interference delivery in murine IRI and unilateral ureteral obstruction models, achieving robust anti-inflammatory and antifibrotic effects. Similarly, Li et al. [50] reported that fibroblastic-reticular-cell-derived EVs naturally homed to injured kidneys and, when enriched with CD5L and further modified with a tubular targeting peptide, substantially improved renal function and survival in septic AKI. These findings support the feasibility of loading antagomiR-491-3p or SIRT1 activators into engineered, kidney-directed EVs, as illustrated in our schematic (Fig. 8M). Thus, EV engineering represents a promising avenue to enhance renal targeting, minimize off-target uptake, and translate EV-based therapies into clinically viable interventions for AKI.

This finding supports the translational potential of applying similar strategies to TEC-derived EV-carried miR-491-3p in IRI treatment. Our findings support further development of EV- or miRNA-targeted therapies and provide a framework for future studies exploring their stability, biodistribution, and clinical feasibility in AKI treatment.

In conclusion, TEC-derived EV-carried miR-491-3p stabilizes NICD by inhibiting SIRT1-mediated deacetylation, enhancing M1 macrophage polarization. This activates the Notch/NF-κB pathway, amplifying inflammation in IRI. Targeting this pathway could reduce inflammation and protect kidney function post-IRI.

## Methods

### Animal model

Male C57BL/6 mice (6 to 8 weeks old) and Rab27a KO mice were used for renal IRI models. IRI was induced by clamping the left renal artery for 45 min (the right kidney was not resected), with tissue collection at 1, 3, and 7 d post-IRI. All procedures followed approved ethical guidelines.

### Cell culture and EV isolation

TCMK1 cells were cultured under hypoxic conditions (5% CO_2_, 94% N_2_, and 1% O_2_) for 24 h, followed by reoxygenation (5% CO_2_, 74% N_2_, and 21% O_2_) for 24 h. EVs were isolated and characterized using nanoparticle tracking analysis and TEM. Raw264.7 macrophages were treated with EVs from TCMK1 cells.

### Molecular and cellular analyses

Western blotting, qPCR, flow cytometry, and dual-luciferase assays were used to analyze protein expression, apoptosis, and gene interactions. Co-IP and ELISA were performed to examine protein interactions and inflammatory cytokines.

### Data statistical analysis

Statistical analyses were conducted using SPSS software version 22.0 (SPSS Inc.). The Shapiro–Wilk test was employed to evaluate the normality of data distribution. For comparisons between 2 groups, the Student *t* test was applied to normally distributed data, whereas the Mann–Whitney *U* test was utilized for nonnormally distributed data. For comparisons among 3 or more groups, one-way analysis of variance followed by the Tukey–Kramer post hoc test was performed. All *P* values were 2-tailed, with values below 0.05 considered statistically significant. Data are presented as mean ± standard deviation.

### Additional methods

All procedural details can be found in the Supplementary Materials.

## Ethical Approval

All animal experiments in this study were reviewed and approved by the Animal Welfare and Ethics Review Committee of Renmin Hospital of Wuhan University (Approval No. WDRM20200308A). All procedures involving animals were performed in strict accordance with institutional guidelines and the National Institutes of Health Guide for the Care and Use of Laboratory Animals. The study did not involve any human participants, human data, or human tissue. Therefore, ethics approval and consent to participate for human subjects were not required.

## Data Availability

The datasets supporting the conclusions of this article are included within the article and its supplementary materials. Additional data, including raw sequencing data and experimental results, are available from the corresponding authors upon reasonable request.
