# Supplementary material for: Tubular-Cell-Derived Extracellular Vesicle miR-491-3p Aggravates Renal Ischemia–Reperfusion Injury by Inhibiting Macrophage SIRT1-Mediated Notch Intracellular Domain Deacetylation-Driven Ubiquitin–Proteasome Degradation
Source: Research (Wash D C). 2025 Oct 17;8:0929. doi: 10.34133/research.0929 (PMC12531491; doi:10.34133/research.0929)
Supplement: Supplementary 1 — Figs. S1 to S18 Tables S1 to S3 [file research.0929.f1.docx]

**Supplementary Figures:**


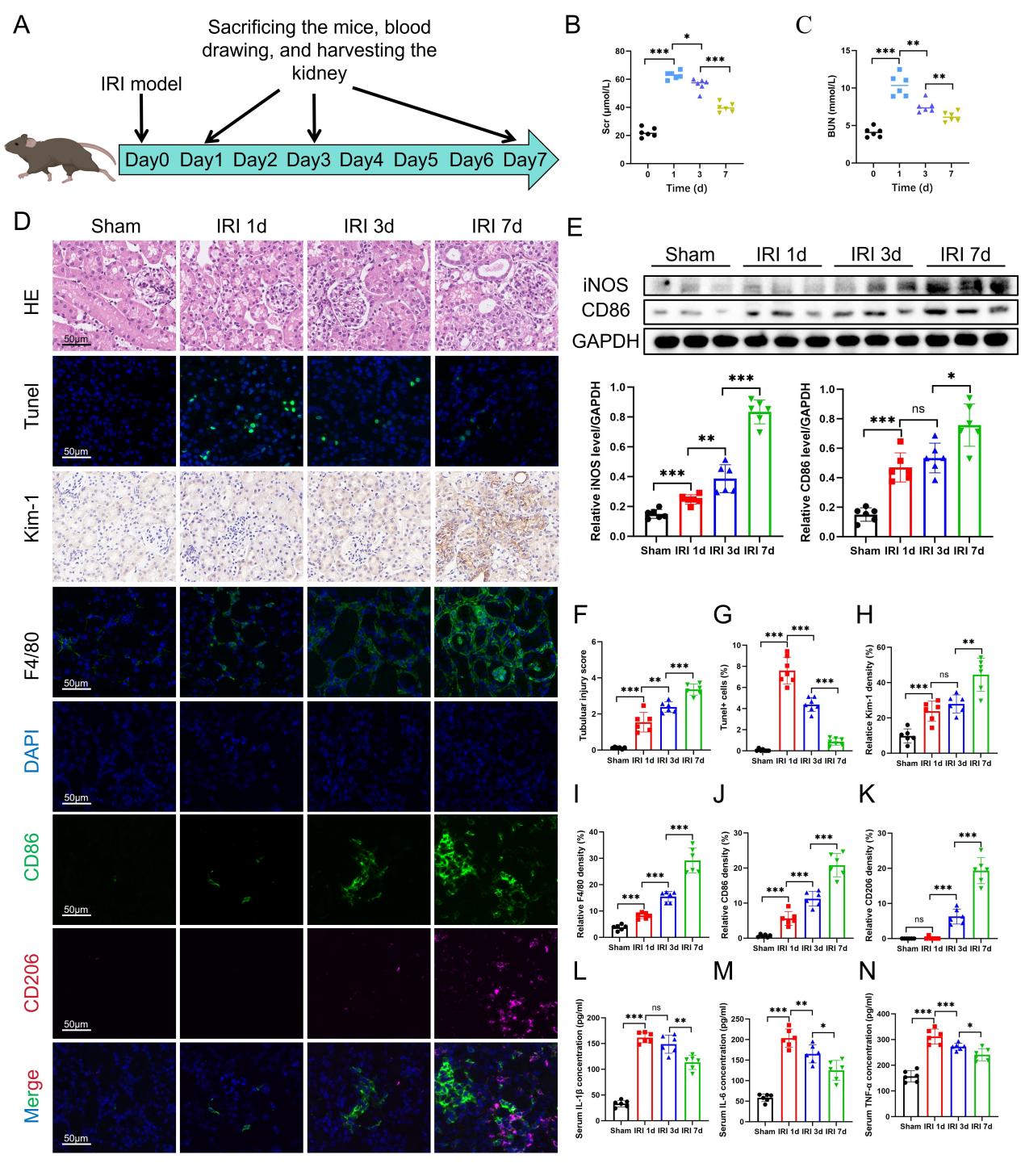


**Supplementary Figure S1.** Construction of the renal IRI model and characterization of macrophage polarization in kidney tissue. (A) A schematic illustration of the renal IRI model in mice and the experimental setup. (B-C) At various intervals, the levels of serum creatinine (B) and urea nitrogen (C) were assessed (n=6). (D) HE and TUNEL staining were used to evaluate renal damage. According to immunohistochemistry, tubular damage was indicated by the expression of Kim-1. Immunofluorescence (IF) was used to visualize macrophage infiltration (F4/80). Double-labeled IF was used to identify the polarization markers of M1 (CD86) and M2 (CD206) macrophages. (E) Western blot analysis was used to determine the protein levels of M1 polarization markers (CD86 and iNOS) (n=6). (F) HE staining was used to assess tubular damage scores (n=6). (G) TUNEL-positive cell count to measure apoptosis (n=6). (H) Immunohistochemical measurement of Kim-1 expression (n=6). (I) Immunofluorescence measurement of macrophage infiltration (F4/80) (n=6). (J-K) Immunofluorescence-based quantification of the percentage of M1 (CD86) (J) and M2 (CD206) (K) macrophages (n=6). (P-R) ELISA was used to assess the serum levels of the pro-inflammatory cytokines TNF-α (R), IL-1β (P), and IL-6 (Q) (n=6). *P < 0.05; **P < 0.01; ***P < 0.001.


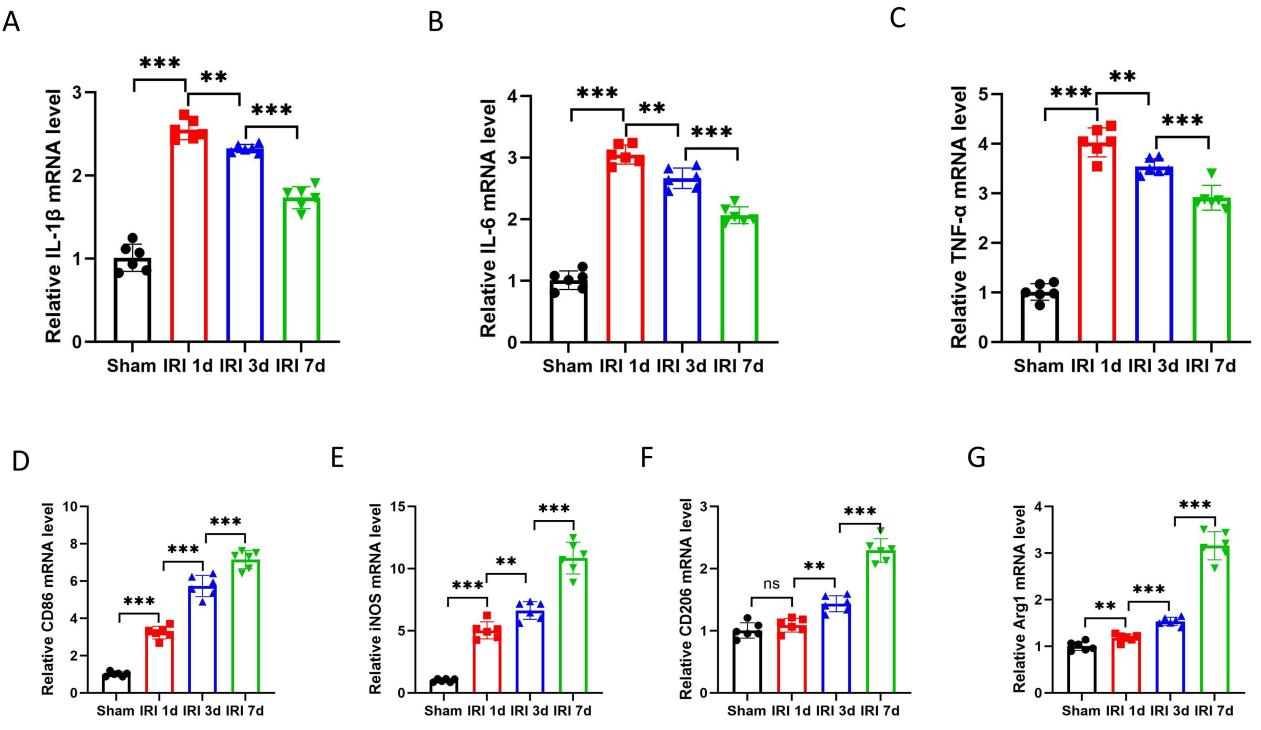


**Supplementary Figure S2.** (A-C) The mRNA levels of inflammatory factors IL-1β, IL-6, and TNF-α in renal tissue at 1, 3, and 7 days after renal ischemia-reperfusion. (D-E) The mRNA levels of macrophage M1 polarization markers CD86 and iNOS in renal tissue at 1, 3, and 7 days after renal ischemia-reperfusion. (F-G) The mRNA levels of macrophage M2 polarization markers CD206 and Arg1 in renal tissue at 1, 3, and 7 days after renal ischemia-reperfusion. *P < 0.05; **P < 0.01; ***P < 0.001.


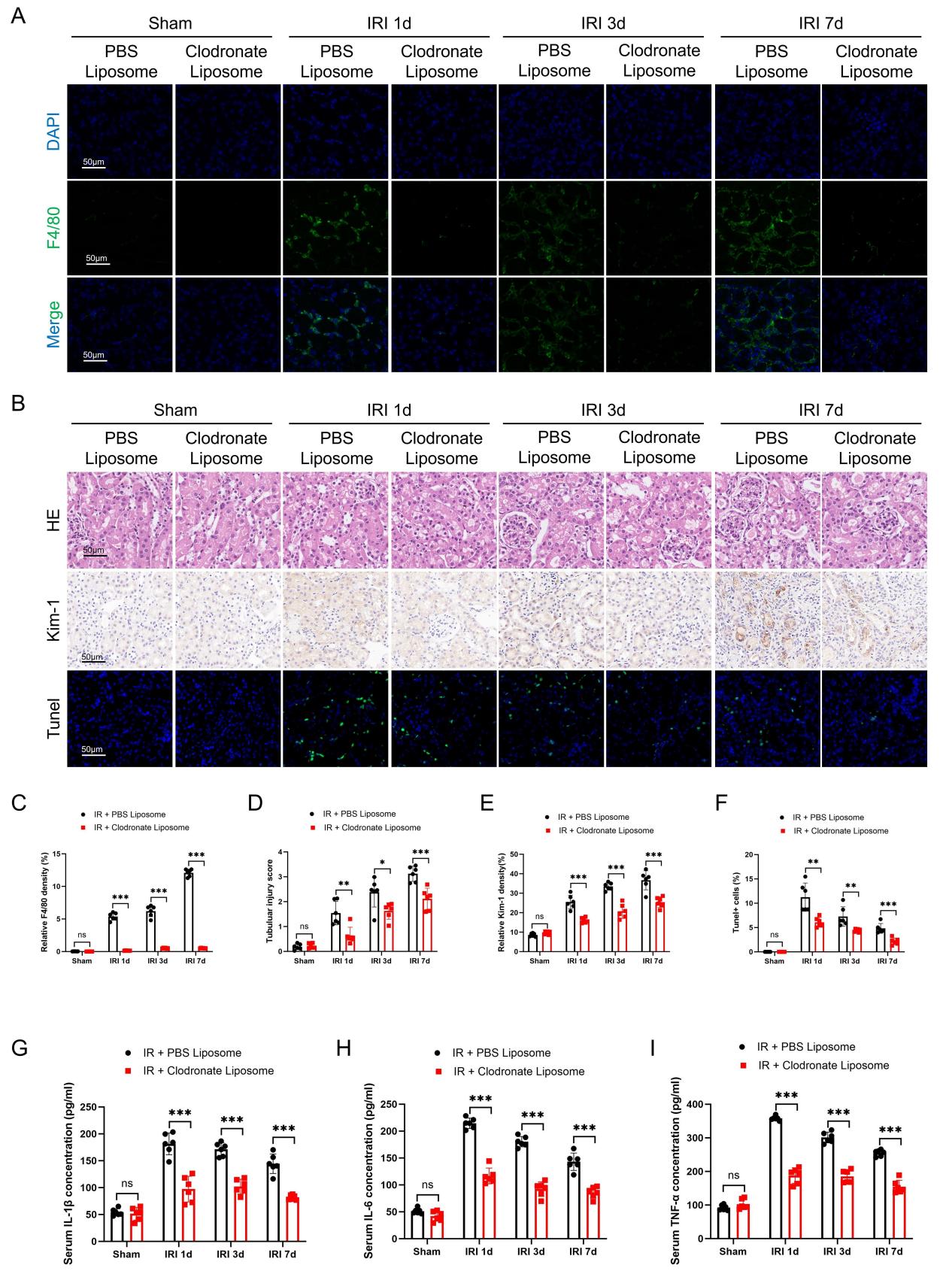


**Supplementary Figure S3.** (A) Representative immunofluorescence staining of kidney sections showing DAPI (blue) for nuclei and F4/80 (green) for macrophage infiltration in Sham, IRI 1d, IRI 3d, and IRI 7d groups treated with PBS liposomes or clodronate liposomes. Macrophage depletion is evident in clodronate-treated groups. (B) Representative histological and immunohistochemical staining of kidney tissues. Hematoxylin and eosin (HE) staining shows tubular injury across groups. Kim-1 immunohistochemistry highlights tubule injury. TUNEL staining (green) demonstrates apoptotic cell death. Macrophage depletion with clodronate liposomes reduces tubular damage and apoptosis compared to PBS liposome groups. (C) Quantification of relative F4/80 positive staining (%) in kidney sections, demonstrating significant macrophage depletion in clodronate liposome-treated groups. (D) Tubular injury scores show increased damage in PBS liposome-treated IRI groups, which is alleviated with clodronate liposomes. (E) Relative Kim-1 expression levels indicate reduced tubular injury in clodronate liposome-treated mice compared to PBS-treated controls. (F) Quantification of TUNEL-positive cells (%) shows a significant reduction in apoptotic cells in clodronate liposome-treated groups. (G-I) ELISA analysis of serum inflammatory cytokines: IL-1β (G), IL-6 (H), and TNF-α (I) concentrations. Macrophage depletion with clodronate liposomes significantly reduces cytokine levels at IRI 1d, IRI 3d, and IRI 7d time points compared to PBS liposome-treated mice. *P < 0.05; **P < 0.01; ***P < 0.001.

**
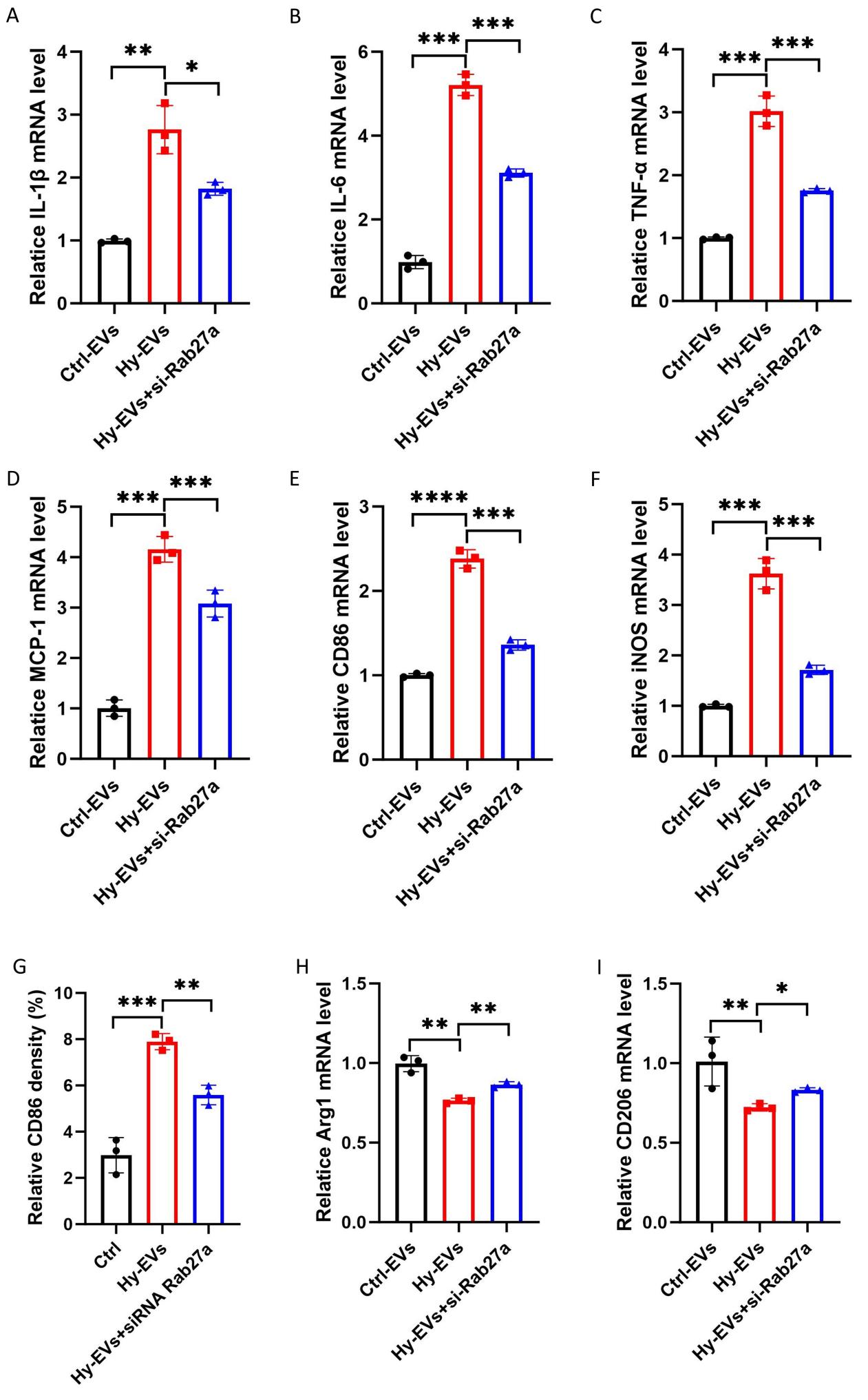
**

**Supplementary Figure S4.** (A-D) The mRNA levels of inflammatory factors IL-1β, IL-6, TNF-α and MCP-1 in the cell culture supernatant of Ctrl-EVs, Hy-EVs and Hy-EVs + si-Rab27a groups. (E) Quantification of CD86 immunofluorescence staining of Raw264.7 cells in different groups (related to Figure 1V). (F-G) The mRNA levels of macrophage M1 polarization markers CD86 and iNOS in the cell culture supernatant of Ctrl-EVs, Hy-EVs and Hy-EVs + si-Rab27a groups. (H-I) The mRNA levels of macrophage M2 polarization markers CD206 and Arg1 in the cell culture supernatant of Ctrl-Exo, Hy-EVs and Hy-EVs + si-Rab27a groups. (n=3). *P < 0.05; **P < 0.01; ***P < 0.001.


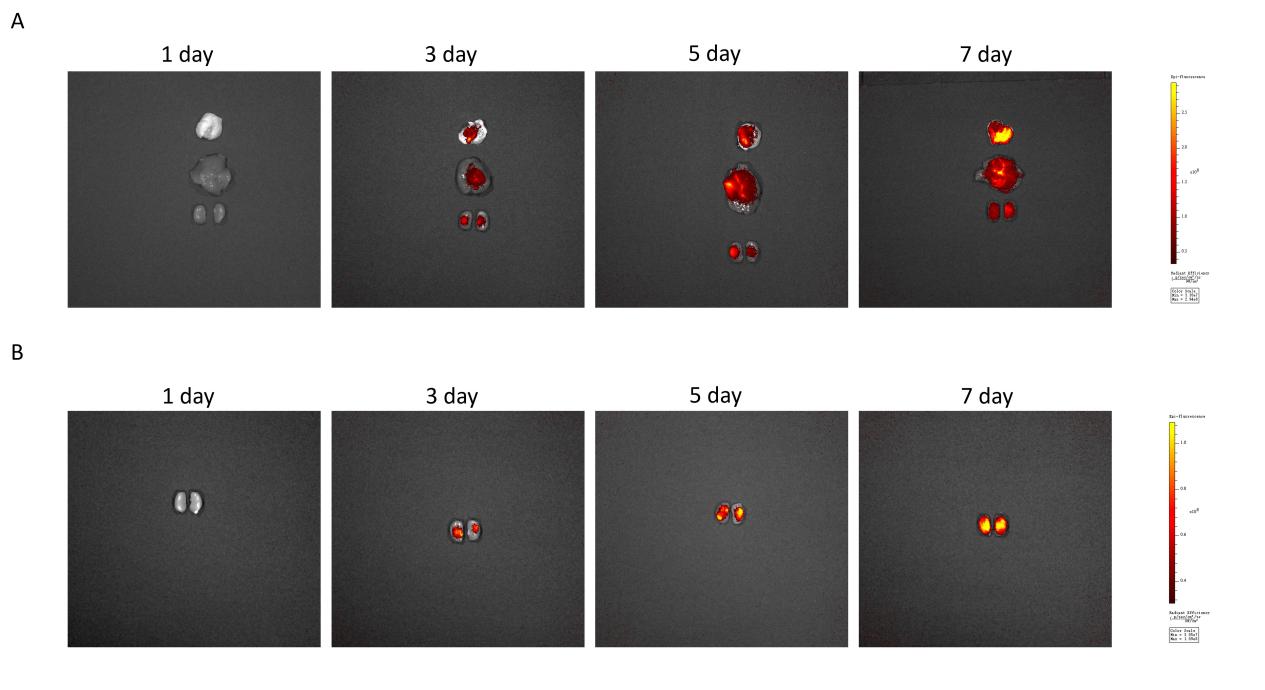


**Supplementary Figure S5.** An IRI model was established, and EVs derived from TCMK1 cells were injected on days 1, 3, and 5. In vivo imaging was conducted on days 1, 3, 5, and 7 to detect DiR-labeled EVs. (A) Fluorescence images of lungs, livers and kidneys. (B) Fluorescence images of kidneys. *P < 0.05; **P < 0.01; ***P < 0.001.


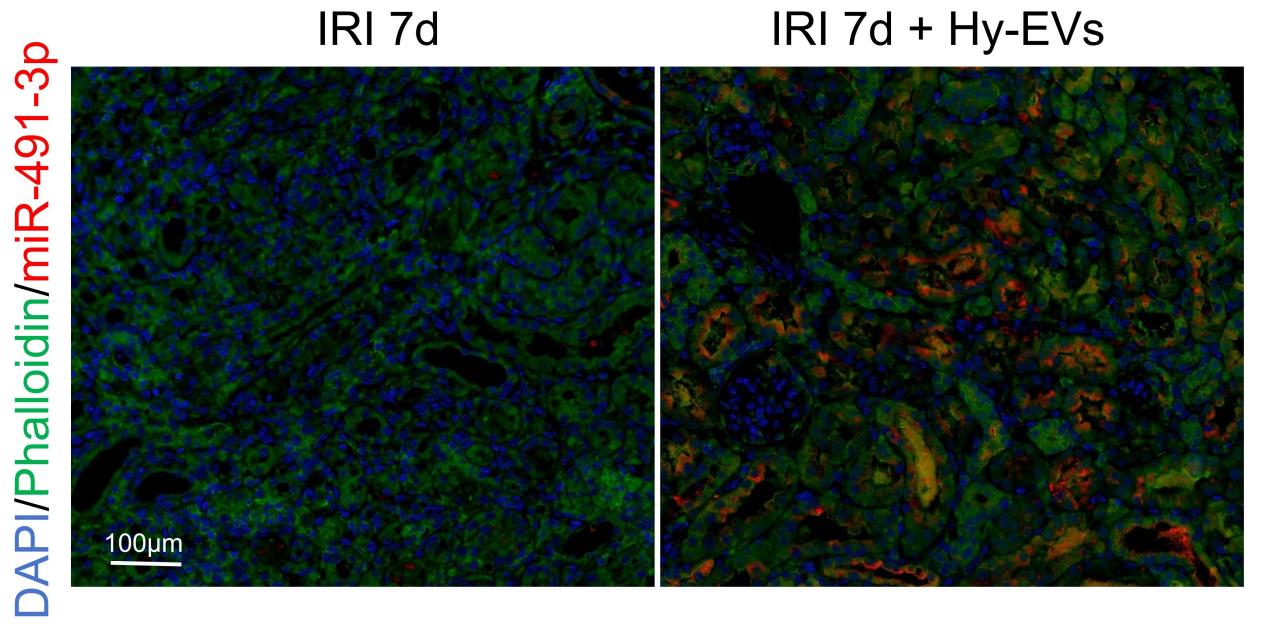


**Supplementaty Figure S6.** Detection of miR-491-3p in kidney tissue by FISH. Representative FISH images of kidney sections from IRI 7d mice and IRI 7d mice injected with Hy-Exo. Red: Cy3-labeled probe for miR-491-3p; Green: Phalloidin (F-actin); Blue: DAPI (nuclei). Compared to the IRI 7d group, Hy-Exo treatment markedly enhanced the accumulation of miR-491-3p signals within renal tubular epithelial cells. Scale bar = 100 μm.


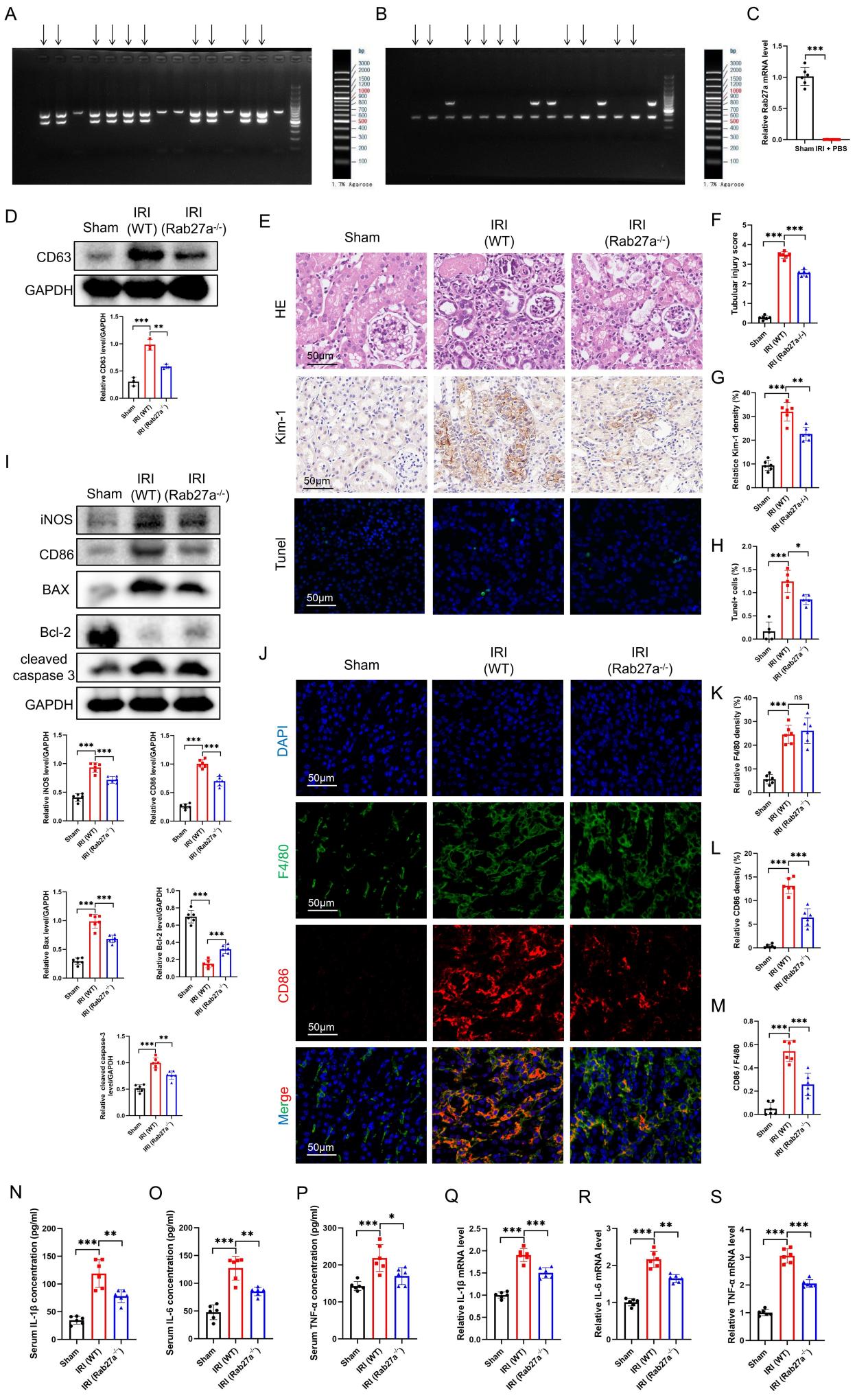


**Supplementary Figure S7.** Rab27a deletion reduces kidney damage in vivo, lowers M1 macrophage polarization, and inhibits EVs' secretion. (A) Rab27a knockout (KO) mice validated by PCR. Rab27a-KO mice are shown by arrows. (B) Rab27a wild-type (WT) mice were subjected to PCR analysis. Rab27a-KO mice are shown by arrows. (C) Comparison of Rab27a mRNA levels in kidney tissues between WT and KO mice. (D) Kidney tissues from Sham, IRI (WT), and IRI (Rab27a-/-) mice (n=6) were subjected to Western blot examination of the EV marker CD63. (E) Samples of kidney tissues from Sham, IRI (WT), and IRI (Rab27a-/-) mice showing HE staining, Kim-1 immunohistochemistry, and TUNEL staining. (F) HE staining-based tubular damage score quantification (n=6). (G) Kim-1 immunohistochemistry quantitative analysis (n=6). (H) Ratio of TUNEL-positive cells in the kidneys of six mice with Sham, IRI (WT), and IRI (Rab27a-/-). (I) Western blot examination of kidney tissues from Sham, IRI (WT), and IRI (Rab27a-/-) mice (n=6) for iNOS, CD86, Bax, Bcl-2, and cleaved caspase-3. (J) Typical pictures of kidney tissue double immunofluorescence staining for F4/80 and CD86. (K-M) Quantitative evaluation of kidney tissues (n=6) for F4/80-positive macrophages (L), CD86-positive macrophages (K), and the CD86 to F4/80 ratio (M). (N-P) Serum levels of IL-1β (N), IL-6 (O), and TNF-α (P) in six mouse groups were measured using ELISA. (Q-S) In kidney tissues from Sham, IRI (WT), and IRI (Rab27a-/-) mice (n=6), the mRNA levels of IL-1β (Q), IL-6 (R), and TNF-α (S) were analyzed by PCR. *P < 0.05; **P < 0.01; ***P < 0.001.


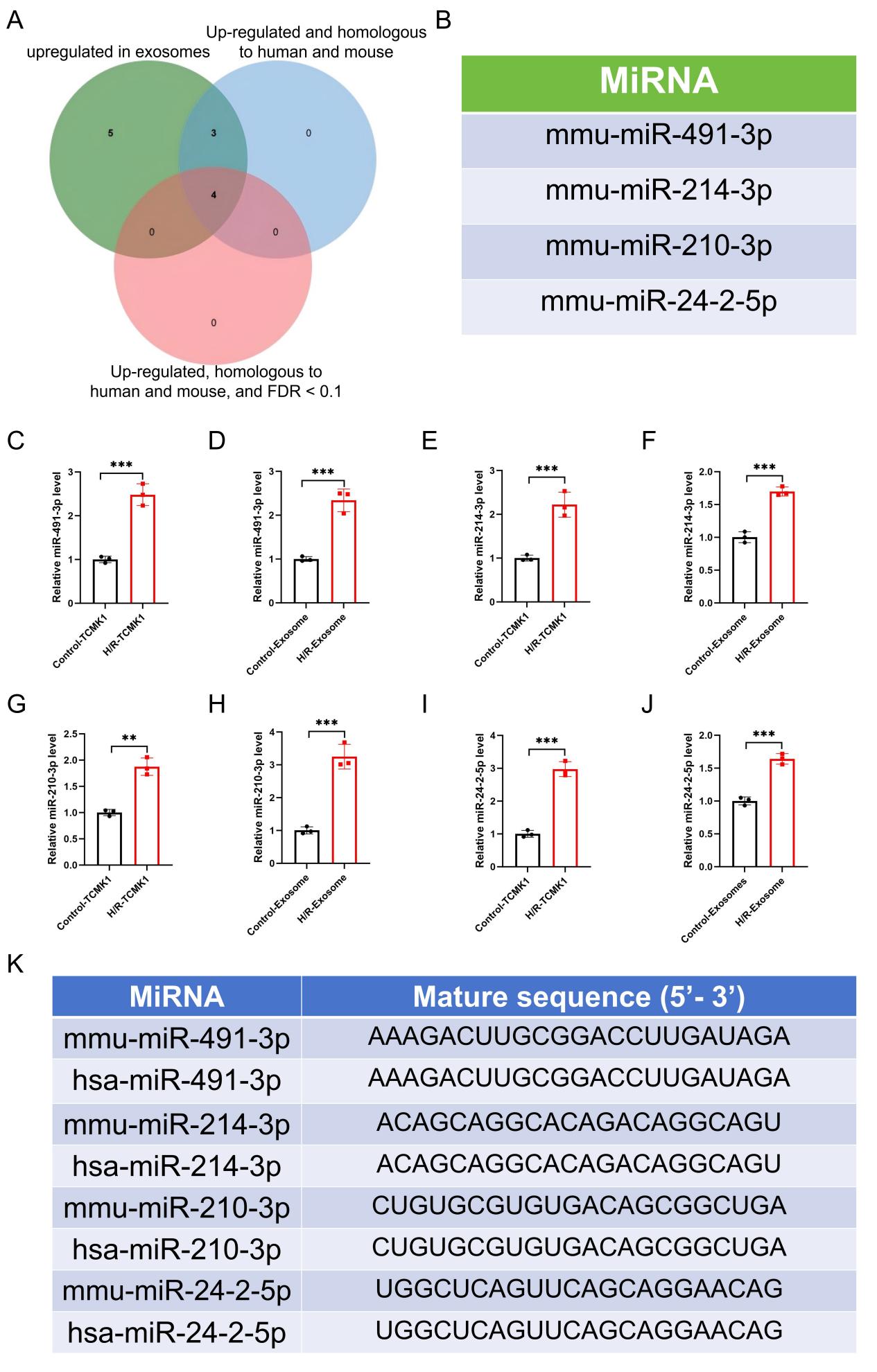


**Supplementary Figure S8.** (A, B) We identified 12 upregulated miRNAs (|Log FC| > 0.5, P < 0.05), including 5 mouse-specific and 7 conserved between humans and mice. Excluding miRNAs with FDR > 0.1, we pinpointed 4 key miRNAs: miR-491-3p, miR-214-3p, miR-210-3p, and miR-24-2-5p. (C, E, G, I) The upregulation multiples of miR-491-3p, miR-214-3p, miR-210-3p, miR-24-2-5p in TCMK1 cells after H/R treatment compared with the control group. (D, F, H, J) The upregulation multiples of miR-491-3p, miR-214-3p, miR-210-3p, miR-24-2-5p in EVs derived from TCMK1 after H/R treatment compared with the control group. (K) Mature sequences of miR-491-3p, miR-214-3p, miR-210-3p, miR-24-2-5 in humans and mice (highly conserved). *P < 0.05; **P < 0.01; ***P < 0.001.


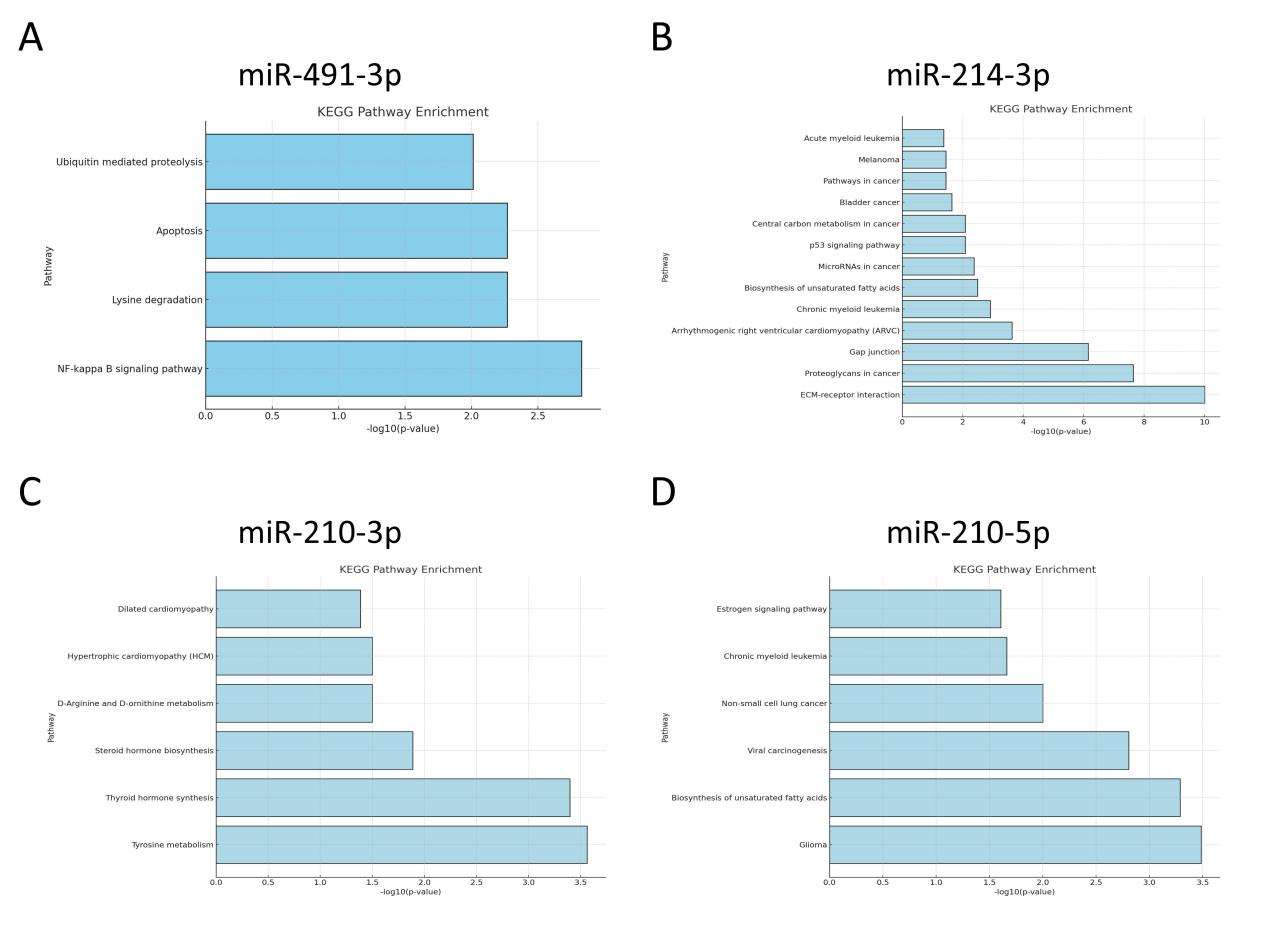


**Supplementary Figure S9.** (A-D) KEGG enrichment analysis of miR-491-3p, miR-214-3p, miR-210-5p, and miR-24-2-5p using the DIANA-miRPath platform revealed significant enrichment of miR-491-3p in pathways related to NF-κB signaling, lysine degradation, apoptosis, and ubiquitin-mediated proteolysis (A). The other three miRNAs were primarily enriched in tumor-related signaling pathways (B-D).


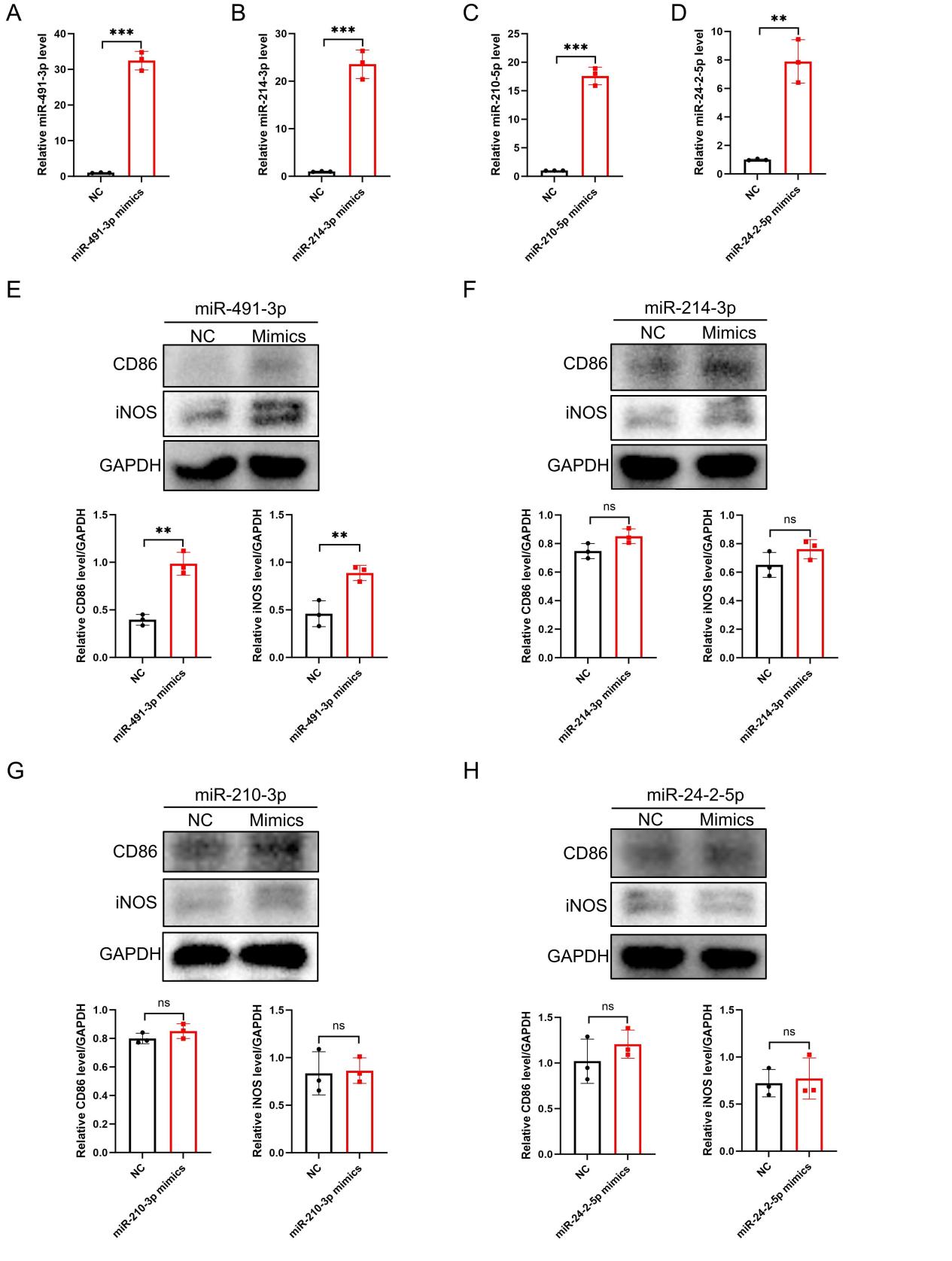


**Supplementary Figure S10.** (A-D) Relative expression levels of miR-491-3p, miR-214-3p, miR-210-3p, and miR-24-2-5p after transfection with their respective mimics in Raw264.7 cells, assessed by qPCR (n = 3). (E-H) Western blot analysis of CD86 and iNOS protein levels, macrophage M1 polarization markers, following transfection with each miRNA mimic (n = 3). Only miR-491-3p mimics significantly upregulated both CD86 and iNOS expression, indicating enhanced M1 polarization. *P < 0.05; **P < 0.01; ***P < 0.001.


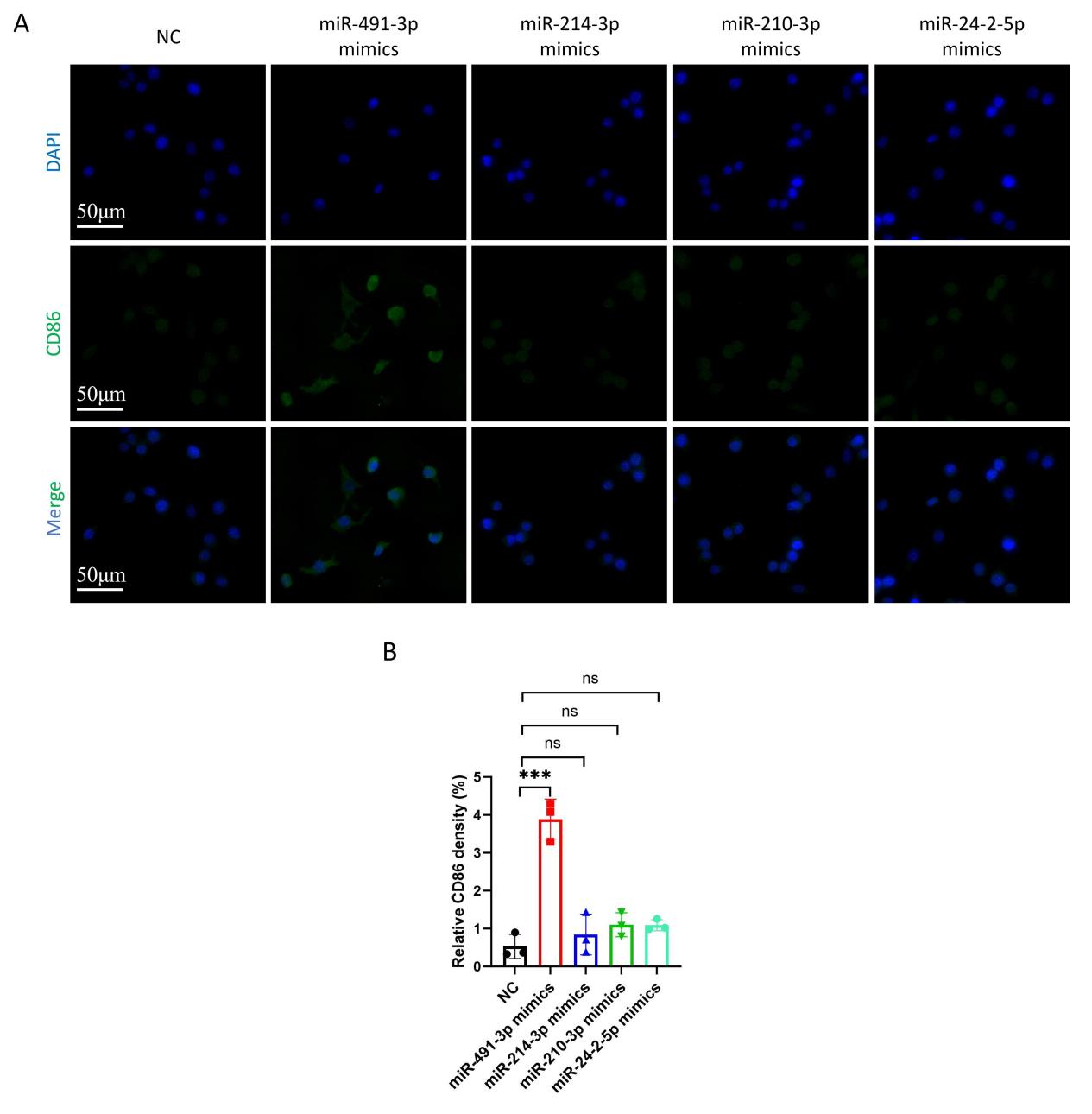


**Supplementary Figure S11.** (A) Representative immunofluorescence images of CD86 (green) in Raw264.7 cells transfected with miR-491-3p, miR-214-3p, miR-210-3p, and miR-24-2-5p mimics, or negative control (NC). Nuclei were stained with DAPI (blue). Scale bar = 50 μm. (B) Quantification of relative CD86 fluorescence intensity. Only miR-491-3p mimics significantly increased CD86 expression compared to NC. *P < 0.05; **P < 0.01; ***P < 0.001.


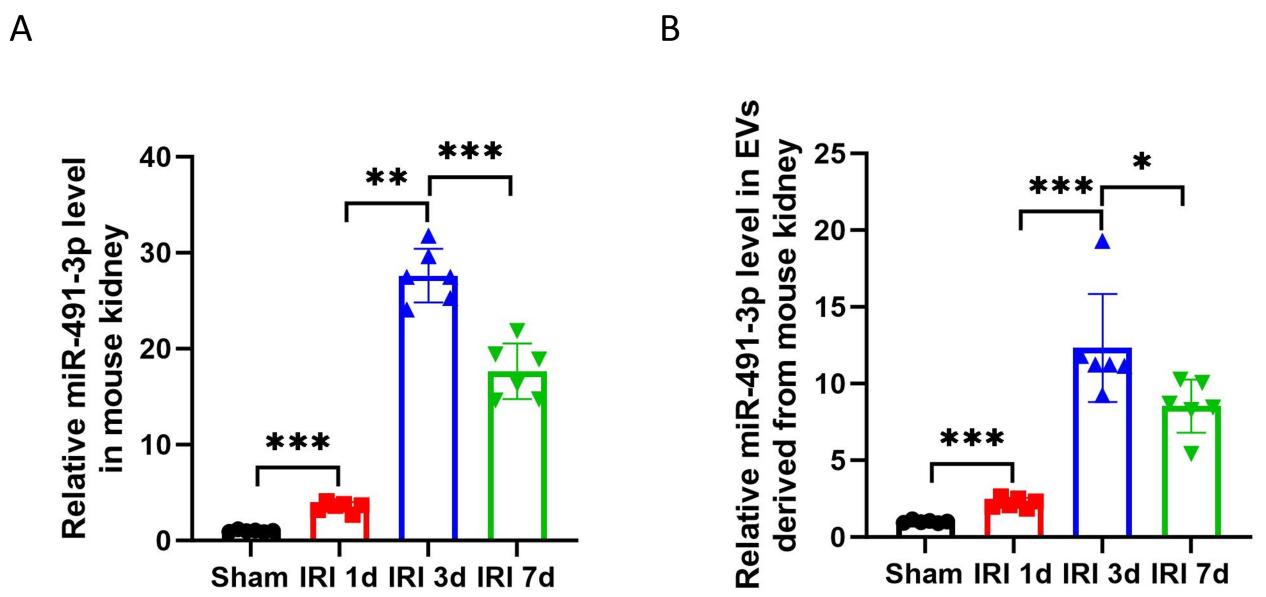


**Supplementary Figure S12.** (A) Relative expression of miR-491-3p in mouse kidney tissue at different time points (Sham, IRI 1 day, IRI 3 days, IRI 7 days), showing a significant increase during the acute phase (1–3 days) and maintaining a high level at 7 days post-IRI. (B) Relative expression of miR-491-3p in EVs derived from mouse kidney at different time points (Sham, IRI 1 day, IRI 3 days, IRI 7 days), showing a significant increase during the acute phase (1–3 days) and maintaining a high level at 7 days post-IRI (n = 6). *P < 0.05, **P < 0.01, ***P < 0.001.


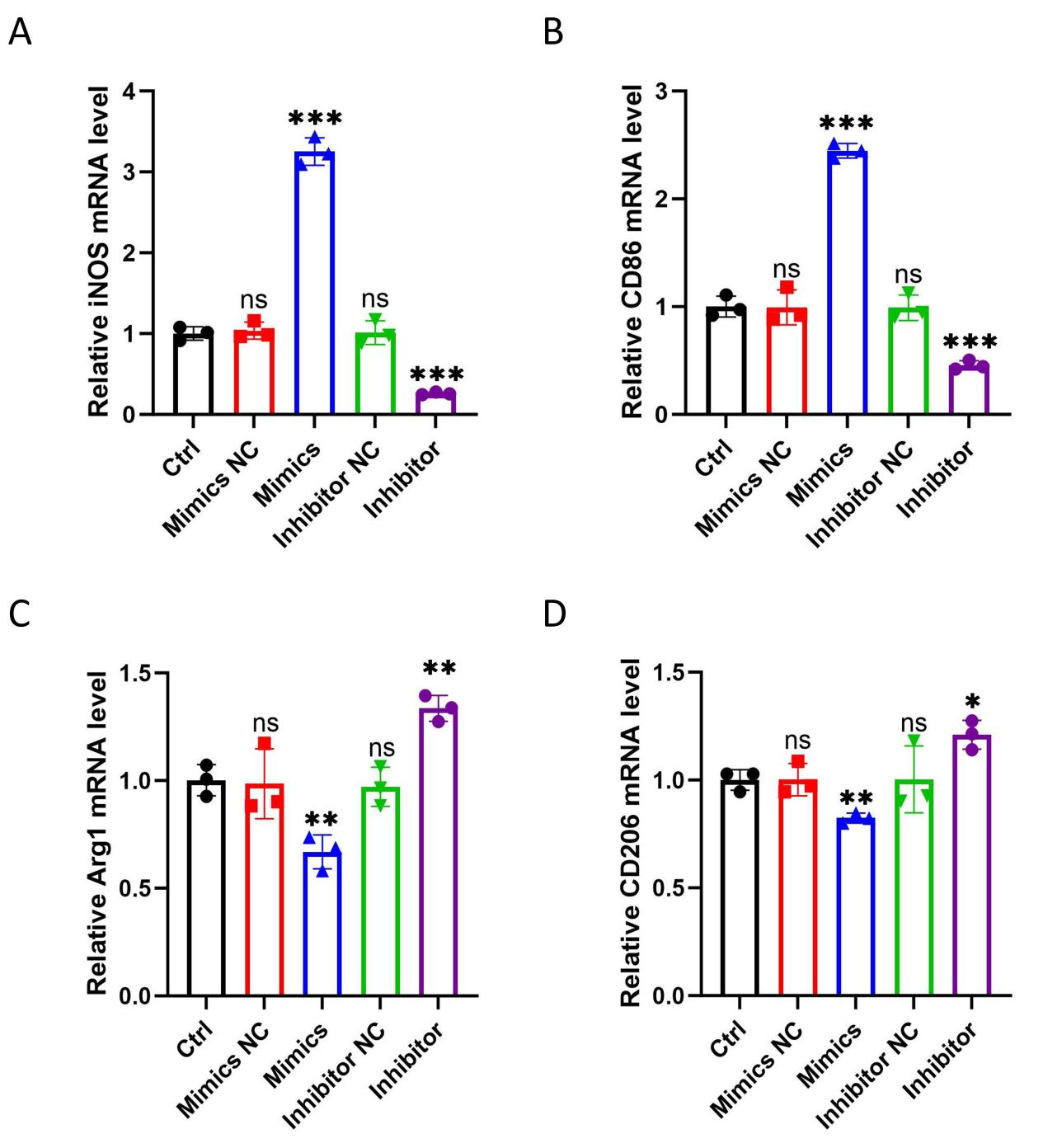


**Supplementary Figure S13.** EV-carried miR-491-3p regulates macrophage polarization in vitro. RAW264.7 macrophages were treated with tubular epithelial cell–derived EVs previously transfected with miR-491-3p mimics or inhibitor. qPCR analysis showed relative mRNA levels of M1 polarization markers (A) iNOS and (B) CD86, and M2 markers (C) Arg1 and (D) CD206. EVs enriched with miR-491-3p significantly increased M1-associated gene expression, whereas EVs from the inhibitor group suppressed M1 markers and promoted M2 marker expression (n = 3). ns, not significant; *P < 0.05; **P < 0.01; ***P < 0.001.


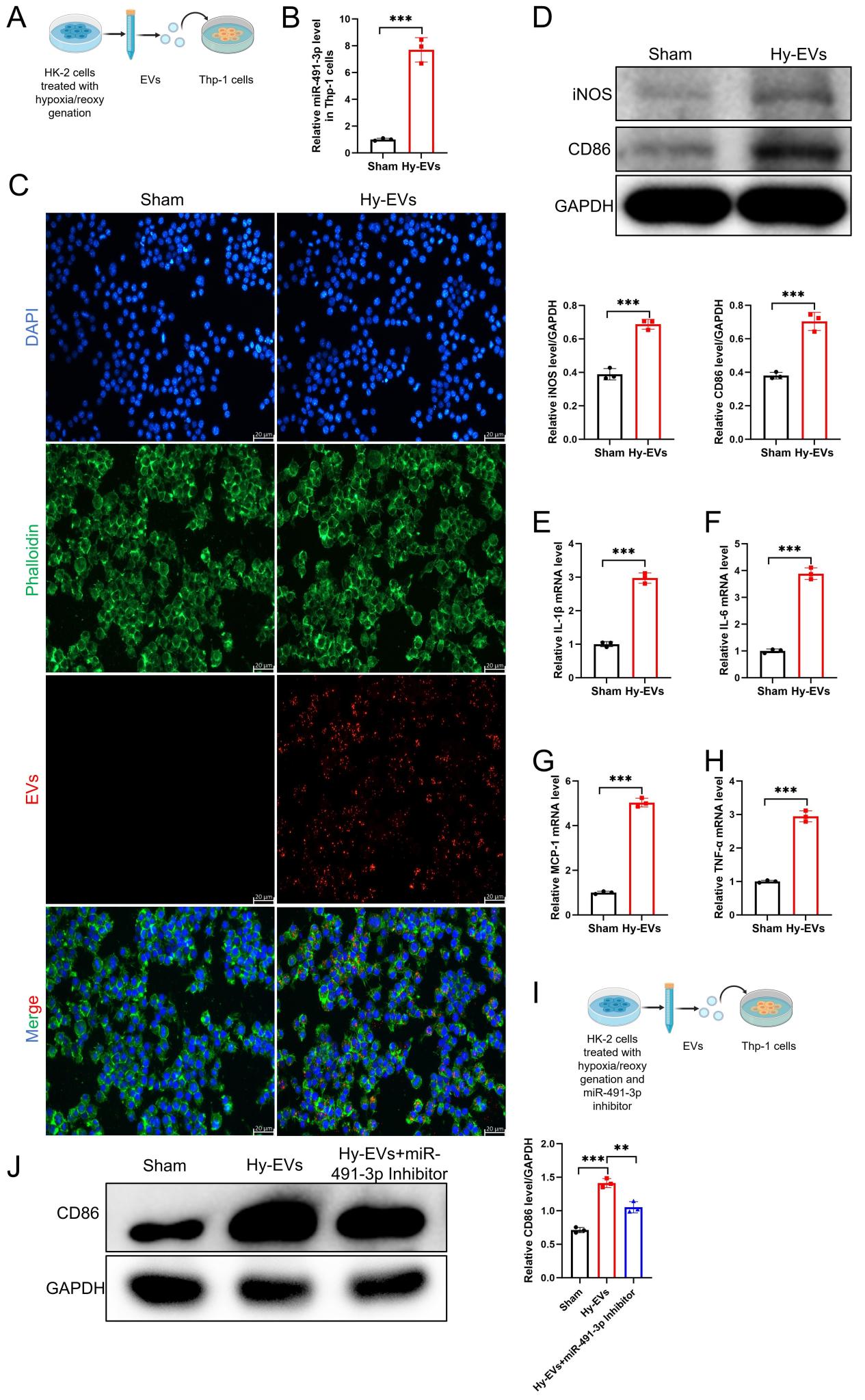


**Supplementary Figure S14.** TEC-derived extracellular vesicles (EVs) transfer miR-491-3p to human macrophage-like cells and promote M1 polarization. (A) Schematic of the experimental design: human renal tubular epithelial HK-2 cells were exposed to hypoxia/reoxygenation (H/R), and EVs were isolated and incubated with human monocytic THP-1 cells. (B) qRT-PCR analysis showing significantly elevated miR-491-3p levels in THP-1 cells after exposure to Hy-EVs compared to sham EVs (n=3). (C) Representative confocal images of THP-1 cells treated with sham or Hy-EVs. Cells were counterstained with DAPI (blue) for nuclei, phalloidin (green) for F-actin, and PKH26-labeled EVs (red) to visualize uptake. Scale bar: 20 μm. (D) Western blot and densitometric quantification demonstrating increased expression of M1 polarization markers iNOS and CD86 in THP-1 cells treated with Hy-EVs (n=3). (E–H) Relative mRNA levels of pro-inflammatory cytokines IL-1β, IL-6, MCP-1, and TNF-α in THP-1 cells were significantly upregulated by Hy-EV treatment (n=3). (I) Experimental schematic showing HK-2 cells treated with H/R, EV isolation, and subsequent treatment of THP-1 cells with or without miR-491-3p inhibitor. (J) Western blot and quantification of CD86 protein expression confirming that inhibition of miR-491-3p attenuated Hy-EV-induced M1 polarization in THP-1 cells (n=3). Data are shown as mean ± SD; *p < 0.05, **p < 0.01, ***p < 0.001.


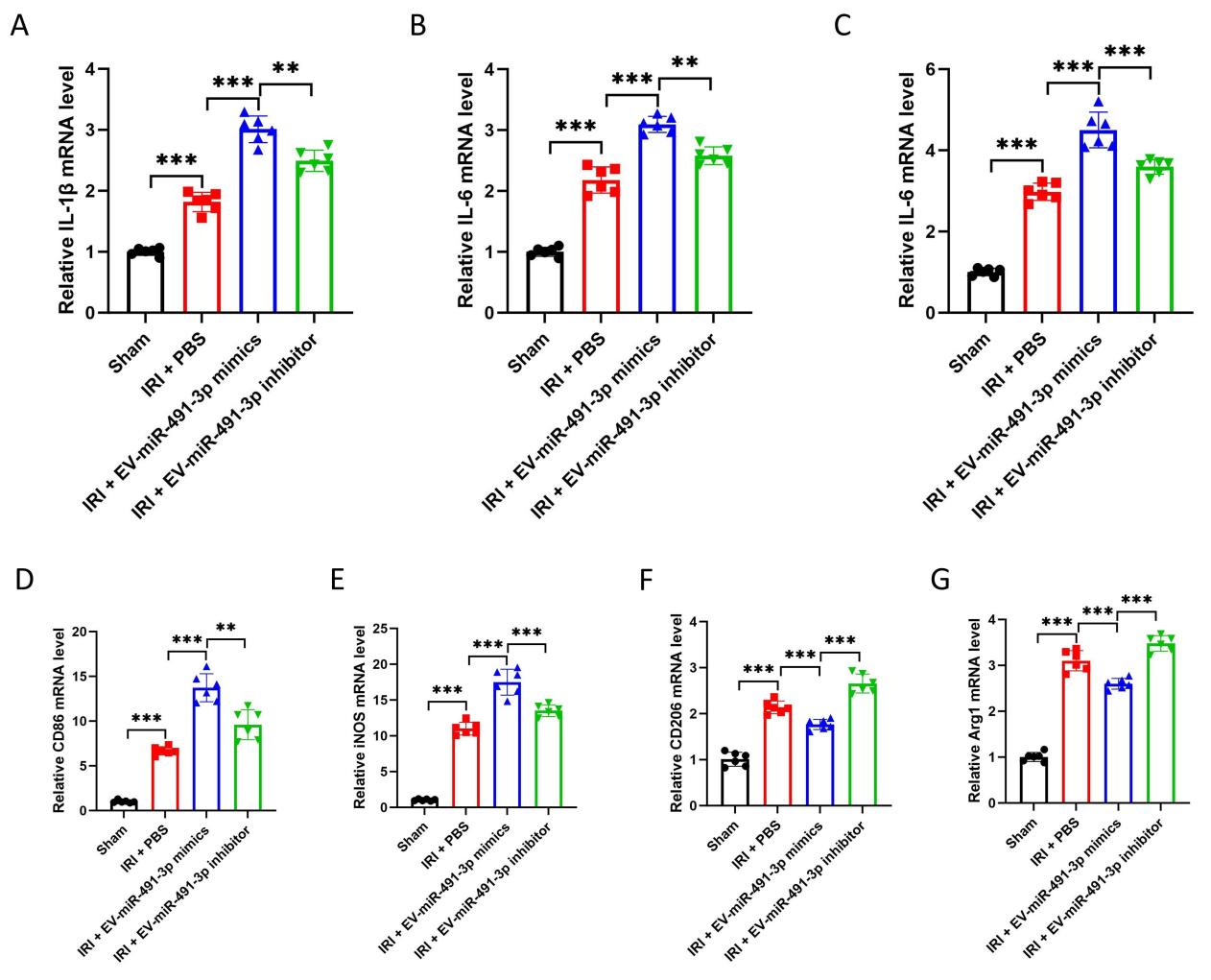


**Supplementary Figure S15.** (A–C) Relative mRNA levels of pro-inflammatory cytokines IL-1β (A), IL-6 (B), and TNF-α (C) in Sham, IRI + PBS, IRI + Exo-miR-491-3p mimics, and IRI + Hy-EV-miR-491-3p inhibitor groups. miR-491-3p mimics significantly upregulated cytokine expression, whereas miR-491-3p inhibition attenuated this effect. (D–G) Relative mRNA levels of Markers of macrophage polarization in Sham, IRI + PBS, IRI + Exo-miR-491-3p mimics, and IRI + Hy-EVs-miR-491-3p inhibitor groups. *P < 0.05; **P < 0.01; ***P < 0.001.


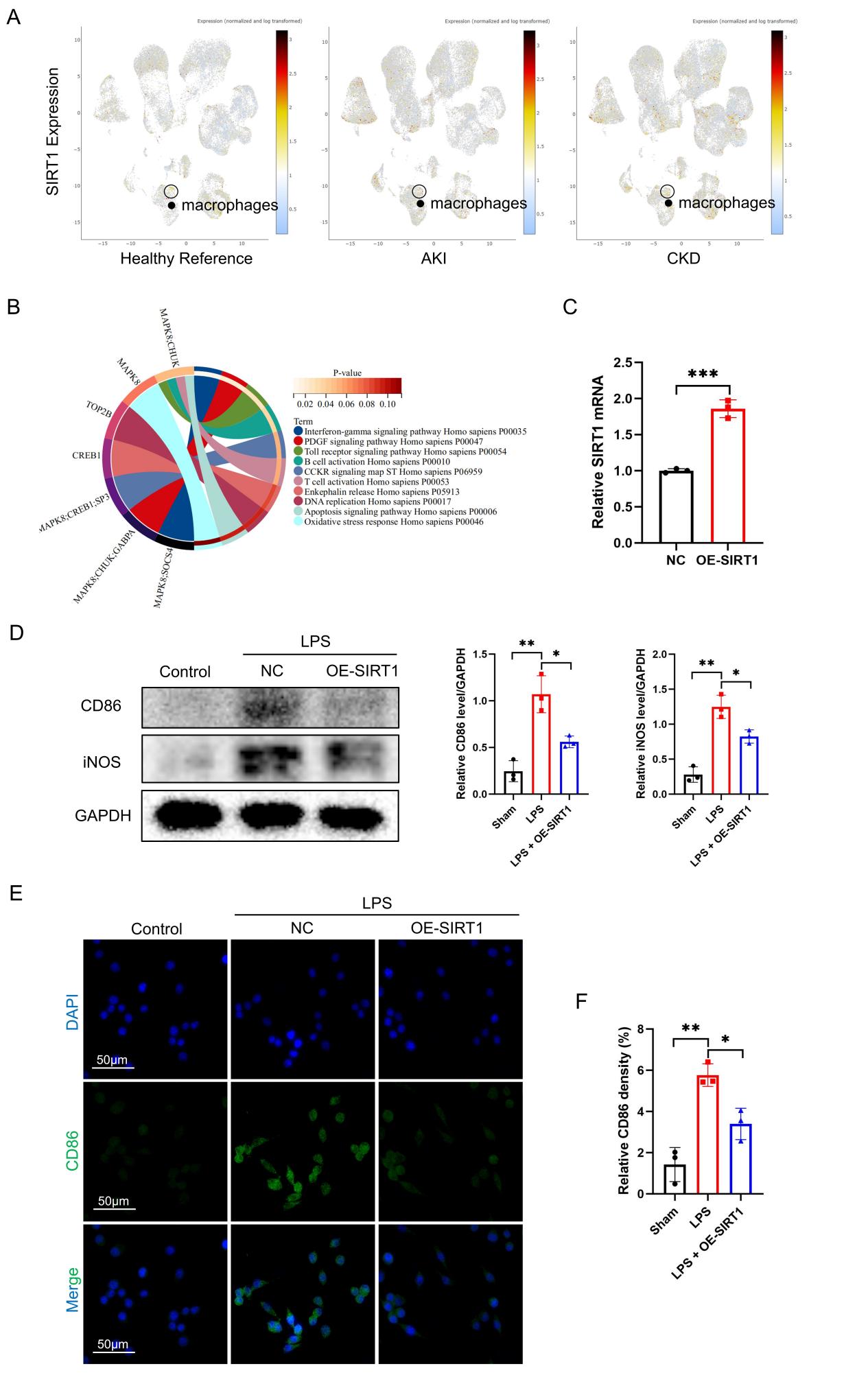


**Supplementary Figure S16.** (A) Single-cell RNA sequencing analysis showing significantly reduced SIRT1 expression in macrophages from kidneys with acute kidney injury (AKI) and chronic kidney disease (CKD) compared to healthy controls. (B) Gene Ontology (GO) enrichment analysis of SIRT1 using the Enrichr database, highlighting its association with inflammation-related pathways such as interferon-gamma signaling and Toll-like receptor signaling. (C) RAW264.7 macrophages were transfected with SIRT1 overexpression plasmid, and PCR analysis confirmed successful upregulation of SIRT1 expression. (D) Western blot analysis of M1 macrophage markers CD86 and iNOS following LPS stimulation with or without SIRT1 overexpression. (E) Immunofluorescence staining of CD86 (green) in macrophages under the same conditions. Scale bar = 50 μm. (F) Quantification of CD86 fluorescence intensity. SIRT1 overexpression significantly attenuated LPS-induced upregulation of CD86 and iNOS, demonstrating its role in suppressing macrophage M1 polarization. *P < 0.05; **P < 0.01; ***P < 0.001.


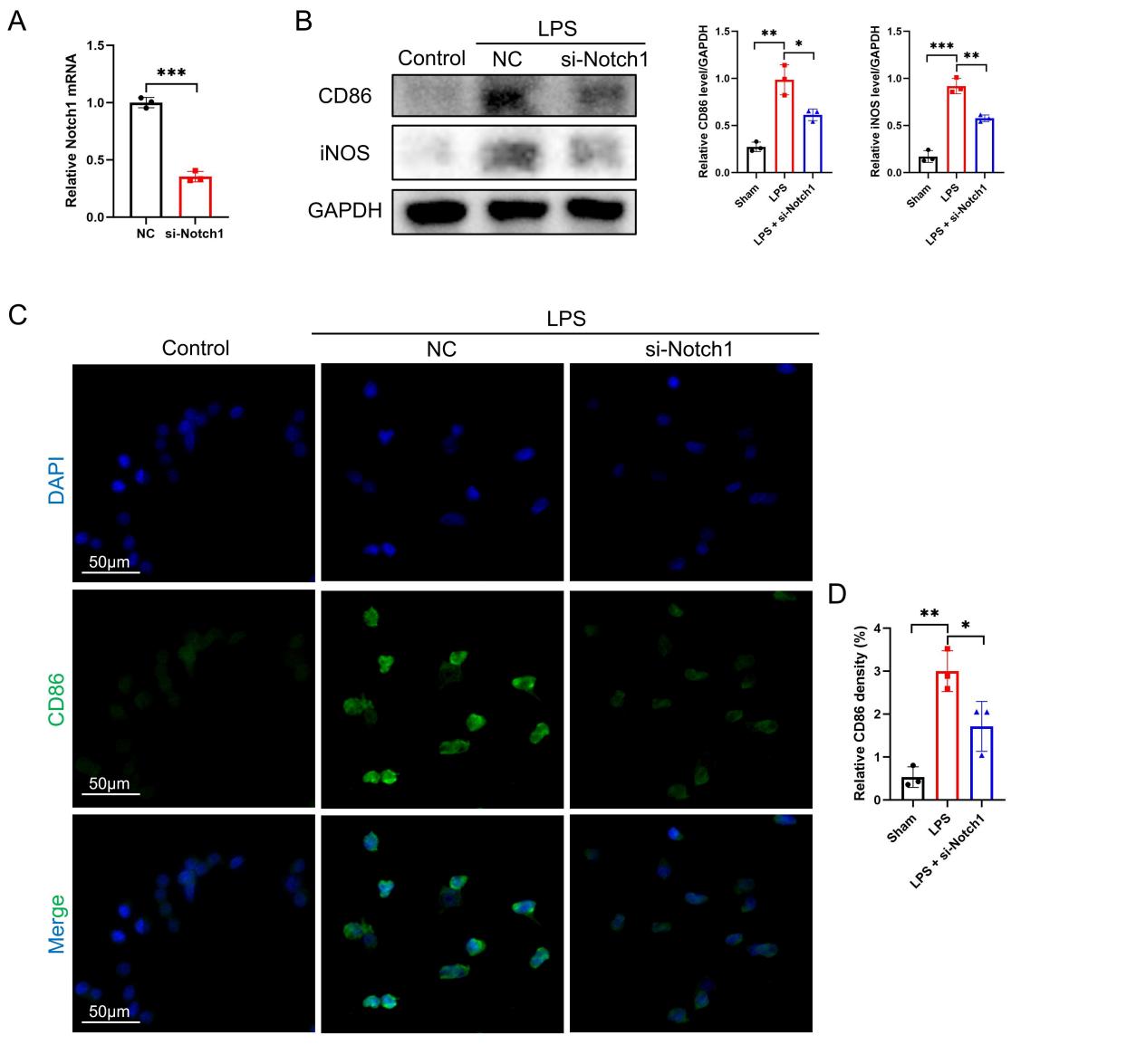


**Supplementary Figure S17.** (A) RAW264.7 macrophages were transfected with si-Notch1 plasmid, and PCR analysis confirmed successful downregulation of Notch1 expression. (B) Western blot analysis of M1 macrophage markers CD86 and iNOS following LPS stimulation with or without Notch1 knockdown. (C) Immunofluorescence staining of CD86 (green) in macrophages under the same conditions. Scale bar = 50 μm. (D) Quantification of CD86 fluorescence intensity. Notch1 knockdown significantly attenuated LPS-induced upregulation of CD86 and iNOS, demonstrating its role in suppressing macrophage M1 polarization. *P < 0.05; **P < 0.01; ***P < 0.001.


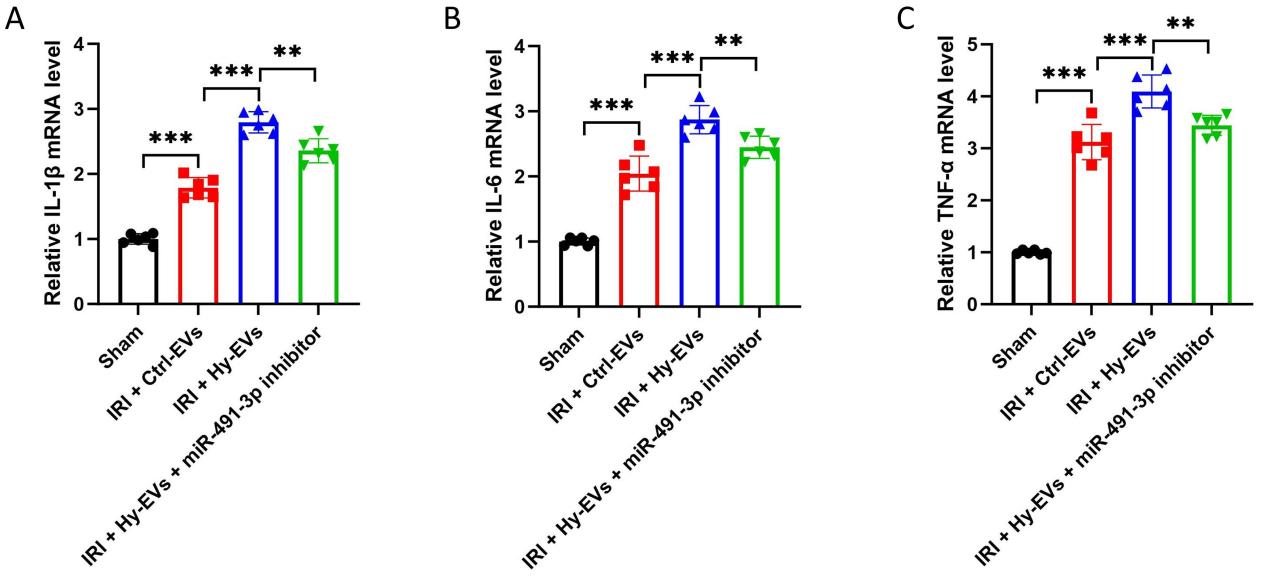


**Supplementary Figure S18.** (A-C) Relative mRNA levels of pro-inflammatory cytokines IL-1β (A), IL-6 (B), and TNF-α (C) in Sham, IRI + Ctrl-EVs, IRI + Hy-EVs, and IRI + Hy-EVs-miR-491-3p inhibitor groups. *P < 0.05; **P < 0.01; ***P < 0.001.

| Agent | Sequence |
| --- | --- |
| miR-491-3p mimic | CUUAUGCAAGAUUCCCUUCUAC |
| miR-214-3p mimic | ACAGCAGGCACAGACAGGCAGU |
| miR-210-5p mimic | CUGUGCGUGUGACAGCGGCUGA |
| miR-24-2-5p mimic | UGGCUCAGUUCAGCAGGAACAG |
| miR-491-3p inhibitor | GUAGAAGGGAAUCUUGCAUAAG |
| si-Rab27a | GGAGAGGUUUCGUAGCUUAUUTT |
| si-Notch1 | GCATGGTGCCGAACCAATACA |
| si-SIRT1 (#1) | CCGUCUCUGUGUCACAAAUTT |
| si-SIRT1 (#2) | GGGAUCAAGAGGUUGUUAATT |
| si-SIRT1 (#3) | CCAAGCAACAAACAACAAUTT |
| si-FBXW7 (#1) | ACCTTCTCTGGAGAGAGAAATGC |
| si-FBXW7 (#2) | GTGTGGAATGCTGAAACTGGAGA |
| si-FBXW7 (#3) | CACAAAGCTGGTGTGCA |

**Supplementary Table S1.** Sequences of miRNAs and siRNAs Used for Transfection in the Study.

| Primer name | Base sequence (5’-3’) |
| --- | --- |
| M-IL-1β |  |
| sense | GGGCCTCAAAGGAAAGAATCT |
| antisense | GAGGTGCTGATGTACCAGTTGG |
| M-IL-6 |  |
| sense | CTGGGAAATCGTGGAAATGAG |
| antisense | AAGGACTCTGGCTTTGTCTTTCT |
| M-TNF-α |  |
| sense | TCCCCAAAGGGATGAGAAGTT |
| antisense | GAGGAGGTTGACTTTCTCCTGG |
| M-MCP-1 |  |
| sense | TAAAAACCTGGATCGGAACCAAA |
| antisense | GCATTAGCTTCAGATTTACGGGT |
| M-SIRT1 |  |
| sense | TTCAGAACCACCAAAGCGG |
| antisense | CAGGAGACAGAAACCCCAG |
| M-Notch1 |  |
| sense | TGGATGGAGACTGCTGGAATG |
| antisense | TAAGGACCTCAAGGCACGGAG |
| M-GAPDH |  |
| sense | TGCTGTCCCTGTATGCCTCTG |
| antisense | TTGATGTCACGCACGATTTCC |
| M-miR-491-3p |  |
| sense | AGGGCTTATGCAAGATTCCC |
| antisense | GTGTGGTGTGGTATGGTGTG |
| H-miR-491-3p |  |
| sense | AGGGCTTATGCAAGATTCCC |
| antisense | GTGTGGTGTGGTATGGTGTG |
| M-miR-214-3p |  |
| sense | TCGCCACAGCAGGCACAGACA |
| antisense | CTCAACTGGTGTCGTGGAGTCGGC |
| M-miR-210-5p |  |
| sense | AGCCACTGCCCACCGC |
| antisense | GAGGAGGAAGAAGAGGAGGA |
| M-miR-24-2-5p |  |
| sense | CGTGCCTACTGAGCTGA |
| antisense | GTTGTGGTTGGTTGGTTTGT |
| M-U6 |  |
| sense | GCTTCGGCAGCACATATACTAAAAT |
| antisense | CGCTTCACGAATTTGCGTGTCAT |
| H-U6 |  |
| sense | GCTTCGGCAGCACATATACTAAAAT |
| antisense | CGCTTCACGAATTTGCGTGT |
| Rab27a-KO |  |
| sense | CATCTCCCTGGTCTCTATAAAATC |
| antisense | ACATCCATAAAACATATTCCCCTC |
| Rab27a-WT |  |
| sense | TAGTGCCTTCTGTCCAACACG |
| antisense | ACATCCATAAAACATATTCCCCTC |
| Rab27a |  |
| sense | GATGGTGGAGAAGCAGTGGG |
| antisense | CTTCAGCAGGTAGTCGTTGTC |

**Supplementary Table S2.** PCR primer sequence.

| Abbreviation | Full Term |
| --- | --- |
| AKI | Acute Kidney Injury |
| IRI | Ischemia-Reperfusion Injury |
| TECs | Tubular Epithelial Cells |
| miRNA | MicroRNA |
| miR-491-3p | microRNA-491-3p |
| EVs | Extracellular Vesicles |
| Hy-EVs | EVs derived from hypoxia/reoxygenation-treated TECs |
| Ctrl-EVs | EVs derived from control TECs |
| NICD | Notch Intracellular Domain |
| SIRT1 | Sirtuin 1 |
| FBXW7 | F-box and WD repeat domain-containing 7 |
| NF-κB | Nuclear Factor kappa-light-chain-enhancer of activated B cells |
| MVB | Multivesicular Body |
| Rab27a | Ras-related protein Rab-27A |
| DAMPs | Damage-Associated Molecular Patterns |
| ROS | Reactive Oxygen Species |
| H/R | Hypoxia/Reoxygenation |
| TCMK1 | Mouse renal tubular epithelial cell line |
| siRNA | Small Interfering RNA |
| Co-IP | Co-Immunoprecipitation |
| WB | Western Blot |
| qPCR | Quantitative Polymerase Chain Reaction |
| ELISA | Enzyme-Linked Immunosorbent Assay |
| TEM | Transmission Electron Microscopy |
| NTA | Nanoparticle Tracking Analysis |
| DLS | Dynamic Light Scattering |
| CHX | Cycloheximide |
| HE | Hematoxylin and Eosin |
| IHC | Immunohistochemistry |
| Scr | Serum Creatinine |
| BUN | Blood Urea Nitrogen |

**Supplementary Figure 3. List of abbreviations.**
